# Pulmonary Neuroendocrine Cells Sense Succinate to Stimulate Myoepithelial Cell Contraction

**DOI:** 10.1016/j.devcel.2022.08.010

**Published:** 2022-09-14

**Authors:** Wenjie Yu, Thomas O. Moninger, Michael Rector, David A. Stoltz, Michael J. Welsh

**Affiliations:** 1 Departments of Internal Medicine Pappajohn Biomedical Institute, Roy J. and Lucille A. Carver College of Medicine, University of Iowa, Iowa City, Iowa 52242 U.S.A.; 2 Molecular Physiology and Biophysics, Pappajohn Biomedical Institute, Roy J. and Lucille A. Carver College of Medicine, University of Iowa, Iowa City, Iowa 52242 U.S.A.; 3 Department of Biomedical Engineering, University of Iowa, Iowa City, Iowa 52242 U.S.A.; 4 Howard Hughes Medical Institute, University of Iowa, Iowa City, Iowa 52242 U.S.A.; 5 Lead contact

**Keywords:** pulmonary neuroendocrine cell, airway submucosal gland, succinate, SUCNR1, cystic fibrosis, myoepithelial cell

## Abstract

Pulmonary neuroendocrine cells (PNECs) are rare airway cells with potential sensory capacity linked to vagal neurons and immune cells. How PNECs sense and respond to external stimuli remains poorly understood. We discovered PNECs located within pig and human submucosal glands, a tissue that produces much of the mucus that defends the lung. These PNECs sense succinate, an inflammatory molecule in liquid lining the airway surface. The results indicate that succinate migrates down the submucosal gland duct to the acinus where it triggers apical succinate receptors causing PNECs to release ATP. The short-range ATP signal stimulates contraction of myoepithelial cells wrapped tightly around the submucosal glands. Succinate-triggered gland contraction may complement action of neurotransmitters that induce mucus release but not gland contraction, to promote mucus ejection onto the airway surface. These findings identify a local circuit in which rare PNECs within submucosal glands sense an environmental cue to orchestrate the function of airway glands.

## eTOC

Yu et al. identify that, in addition to the airway surface, pulmonary neuroendocrine cells (PNECs) localize in submucosal glands of pigs and humans. PNECs express SUCNR1 to sense luminal succinate and trigger ATP release. ATP stimulates myoepithelial cell contraction via P2Y1 receptors, resulting in glandular mucus ejection.

## Graphical Abstract

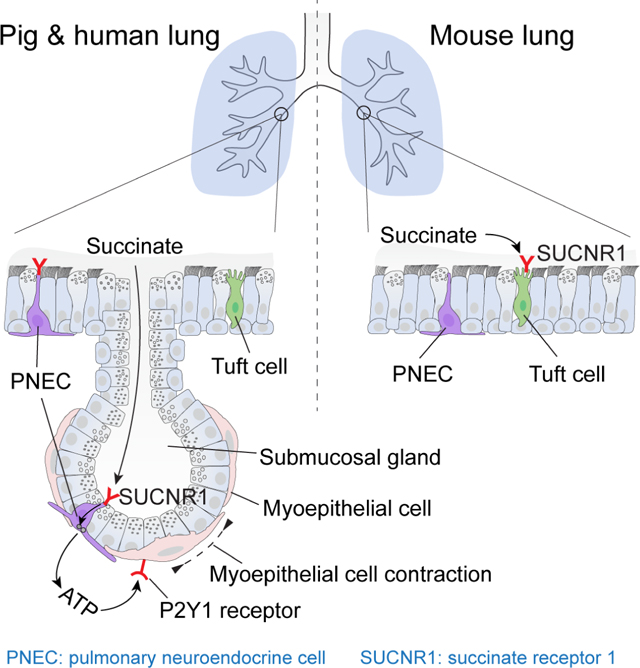

## INTRODUCTION

Neuroendocrine cells are a rare cell type that localizes on the luminal surface of epithelial tissues including the lung and gastrointestinal tract. In the lungs, pulmonary neuroendocrine cells (PNECs) are thought to be exclusively localized to surface epithelia where they are sparsely distributed or clustered at distal airway branch points (Branchfield et al., 2016; Cho et al., 1989; Hogan et al., 2014; Kuo and Krasnow, 2015). They are chemosensory cells that respond to odorants, oxygen, mechanical stimuli, and allergens by releasing neurotransmitters and neuropeptides (Cutz and Jackson, 1999; Gu et al., 2014; Lembrechts et al., 2012; Noguchi et al., 2020; Whitsett and Morrisey, 2016; Xu et al., 2020), which signal either parasympathetic afferents (Chang et al., 2015; Liu et al., 2021; Su et al., 2022) or local immune and epithelial cells (Branchfield et al., 2016; Sui et al., 2018; Xu et al., 2022). As examples, PNECs respond to allergens and hypoxia by stimulating mucus cell production and tissue repair by the airway surface epithelium (Shivaraju et al., 2021; Sui et al., 2018). However, PNEC hyperplasia correlates with many human diseases such as PNEC hyperplasia of infancy, sudden infant death syndrome, asthma, chronic obstructive pulmonary disease, and cystic fibrosis (CF) (Noguchi et al., 2020; Xu et al., 2020). Although our understanding of PNECs has advanced, how PNECs sense and respond to luminal stimuli remains poorly understood.

An opportunity to investigate PNEC-related chemosensation and function arose when we conducted single-cell transcriptome profiling of porcine airway submucosal glands (SMGs) and discovered PNECs (Yu et al., 2022). In large airways of humans and other large mammals, SMGs secrete most of the mucus, which is required to protect the lungs from inhaled and aspirated microorganisms and pollutants (Fahy and Dickey, 2010; Ostedgaard et al., 2020; Widdicombe and Wine, 2015; Wine and Joo, 2004). However, mucus produced in abnormal amounts or with aberrant properties initiates and worsens lung diseases (Fahy and Dickey, 2010; Stoltz et al., 2015; Widdicombe and Wine, 2015). Thus, regulating SMG function is critical in health and disease. Previous studies have shown that vagal parasympathetic efferents and cholinergic agonists stimulate mucus secretion from SMG acinar cells and induce SMG contraction (Ianowski et al., 2007; Widdicombe and Wine, 2015; Wine, 2007). Other neurohumoral agents including substance P and vasoactive intestinal polypeptide stimulate mucus secretion from acinar cells but do not cause SMG contraction (Choi et al., 2007; Choi et al., 2009; Khansaheb et al., 2011). Inflammatory cells also influence SMG function (Kishioka et al., 2001; Lundgren et al., 1991; Widdicombe and Wine, 2015). However, non-neuronal regulatory mechanisms in which SMGs sense the local environment and elicit a response in a restricted area are not well investigated, even though many biological systems possess local control mechanisms.

Finding PNECs in SMGs suggested that they might be a cell type that senses the luminal environment and exerts local control on SMG function. To test this idea, we investigated PNECs in newborn pig SMGs. We chose pigs because their lungs closely mirror the anatomy, physiology, and disease phenotypes of human lungs, including abundant SMGs in the intrapulmonary airways (Choi et al., 2000; Judge et al., 2014; Rogers et al., 2008a). Using newborn animals avoids confounding influences from inflammation, infection, and disease. Our data revealed that PNECs specifically express SUCNR1, the receptor of the inflammatory indicator succinate. We found that succinate activates SUCNR1 on SMG PNECs, causing ATP-P2Y1R-dependent contraction of myoepithelial cells wrapped around SMGs. The results suggest that succinate-mediated gland contraction may complement substance P stimulated mucus secretion to promote mucus ejection from SMGs. These findings identify a submucosal tissue distribution, chemosensory mechanism, and physiological function of PNECs.

## RESULTS

### PNECs are located in pig and human airway SMGs

**Figure 1 |:**
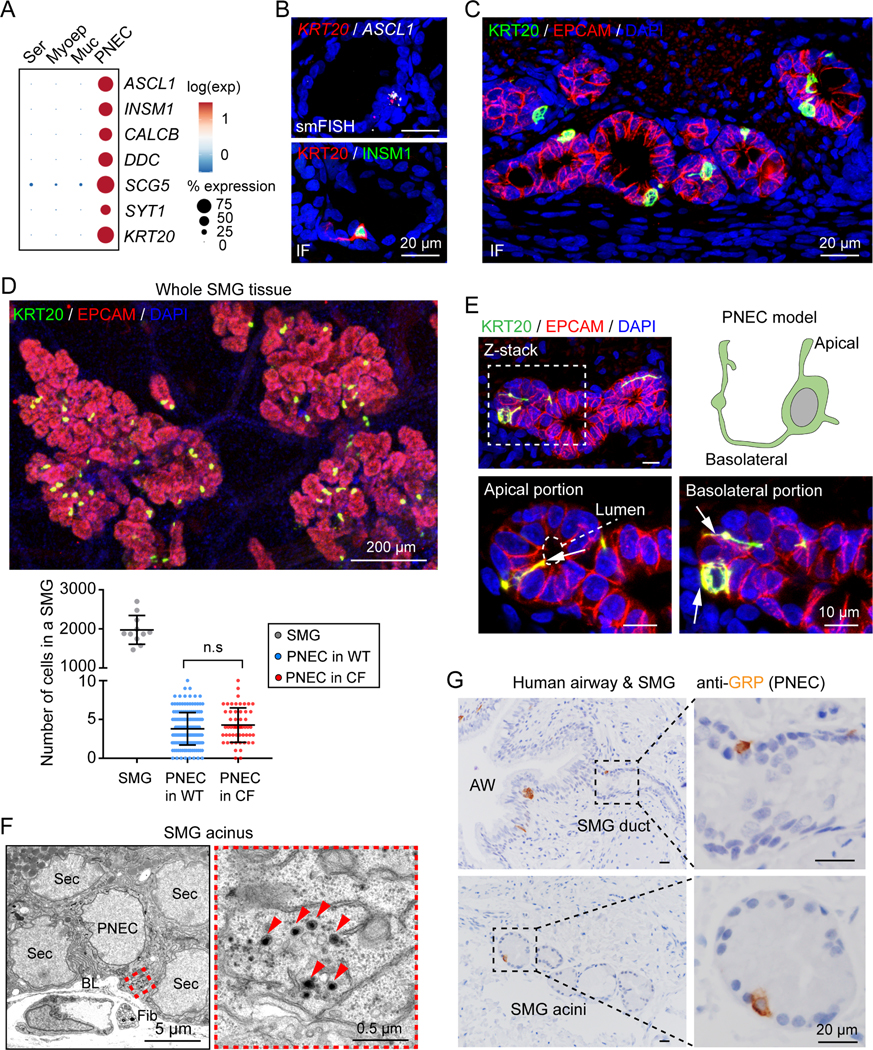
PNECs are localized in porcine and human SMGs. **A)** PNEC marker gene expression in single-cell RNA sequencing (scRNA-seq) data from porcine SMGs (Yu et al., 2022). Ser, serous cell; Myoep, myoepithelial cell; Muc, mucous cell. **B, C)** Single-molecule fluorescence *in situ* hybridization (smFISH) and immunofluorescence (IF) of PNEC markers (ASCL1, INSM1, KRT20) in SMGs (EPCAM+). **D)** Quantification of PNECs in individual SMGs. Upper panel, immunostaining of PNECs (KRT20+, green) in whole mount SMGs (EPCAM+, red). Lower panel, summary of total cells and PNECs in individual SMGs. Each data point represents number of cells or PNECs in a SMG. P value was calculated between WT and CF based on individual SMGs; p > 0.05 is considered as not significant (n.s.), unpaired Student’s *t* test; n = 3–4 pigs. **E)** SMG PNEC reconstruction. Enlarged images show apical and basolateral portions (arrows) of a PNEC connecting to the acinar lumen (dashed outline) and other SMG cells. **F)** Representative transmission electron microscopic images of rare cells (PNECs) with dense core vesicles (arrowheads) in SMGs. Sec, secretory cell of SMGs; BL, Basal lamina; Fib, fibroblast. **G)** Immunohistochemistry of gastrin-releasing peptide (GRP, human PNEC marker) in human airways (AW) and SMGs. See also Figure S1 and Figure S2.

Single-cell transcriptomic analysis of porcine SMGs revealed a cluster of rare epithelial cells that were enriched with key PNEC transcription factors (*ASCL1, INSM1*) and neuroendocrine signature genes (*CALCB, DDC, SCG5, SYT1*) (Figure 1A), indicating that they are PNECs (Jia et al., 2015; Noguchi *et al*., 2020; Sun et al., 2022; Xu et al., 2020). Single-molecule fluorescence *in situ* hybridization (smFISH) and immunostaining revealed representative PNEC markers plus an additional marker *cytokeratin 20* (*KRT20*) (Figures 1B and 1C). We discovered PNECs scattered throughout airway SMGs, including in both the ducts and acini of newborn pigs (Figures 1B, 1C, and S1A). They were also present in SMGs of older pigs (Figure S1B). Using the same markers, we also found scattered PNECs in the surface epithelial layer, consistent with previous reports (Figure S1C).

To estimate the frequency with which PNECs occur in SMGs, we counted total cells and PNECs in SMGs after labeling whole mount newborn tracheal SMGs with an epithelial marker E-cadherin or EPCAM and a PNEC marker KRT20. We found ~2000 total cells and ~4 PNECs (0.2%) per SMG (Figure 1D). This ratio is similar to the ratio of PNEC/total epithelial cells in pig SMG scRNA-seq data (0.3%) and human large airway scRNA-seq data (0.16–0.23%) (Deprez et al., 2020; Goldfarbmuren et al., 2020; Yu et al., 2022). Although PNEC hyperplasia can occur in lung diseases including cystic fibrosis (CF), in newborn wild-type (WT) and CF pigs, PNEC numbers per SMGs did not differ (Figure 1D).

PNECs in SMGs had features that are characteristic of PNECs in airway surface epithelia (Noguchi et al., 2020; Sun et al., 2022; Xu et al., 2020). They had a unique morphology with a central flask-shaped cell body containing the nucleus and single or multiple long, narrow branches that contacted the luminal surface or traveled around and beneath surrounding cells (Figure 1E). Consistent with previous studies of PNECs, we found rare cells in SMGs that had dense core vesicles, which are typical neuroendocrine features of PNECs (Figure 1F) (DiAugustine and Sonstegard, 1984; Yeger et al., 1996). SMG PNECs were also enriched for neurotransmitters and neuropeptides previously reported for PNECs, including calcitonin gene-related peptide (CGRP, the protein of *CALCA* and *CALCB*) and serotonin (5-HT) (Noguchi et al., 2020; Xu et al., 2020) (Figures S1D-S1F). Of note, they did not express transcripts for ion channels such as CFTR or TMEM16A that mediate transepithelial Cl^-^ and HCO_3_^-^ secretion in other SMG cell types (Figure S1G). Like porcine SMGs, human SMGs also contained PNECs in the ductal and acinar region regardless of age and disease (Figures 1G and S2). These data suggest conservation of PNECs in the SMGs of humans and pigs.

### SMG PNECs are chemosensory cells that specifically express the succinate receptor 1

**Figure 2 |:**
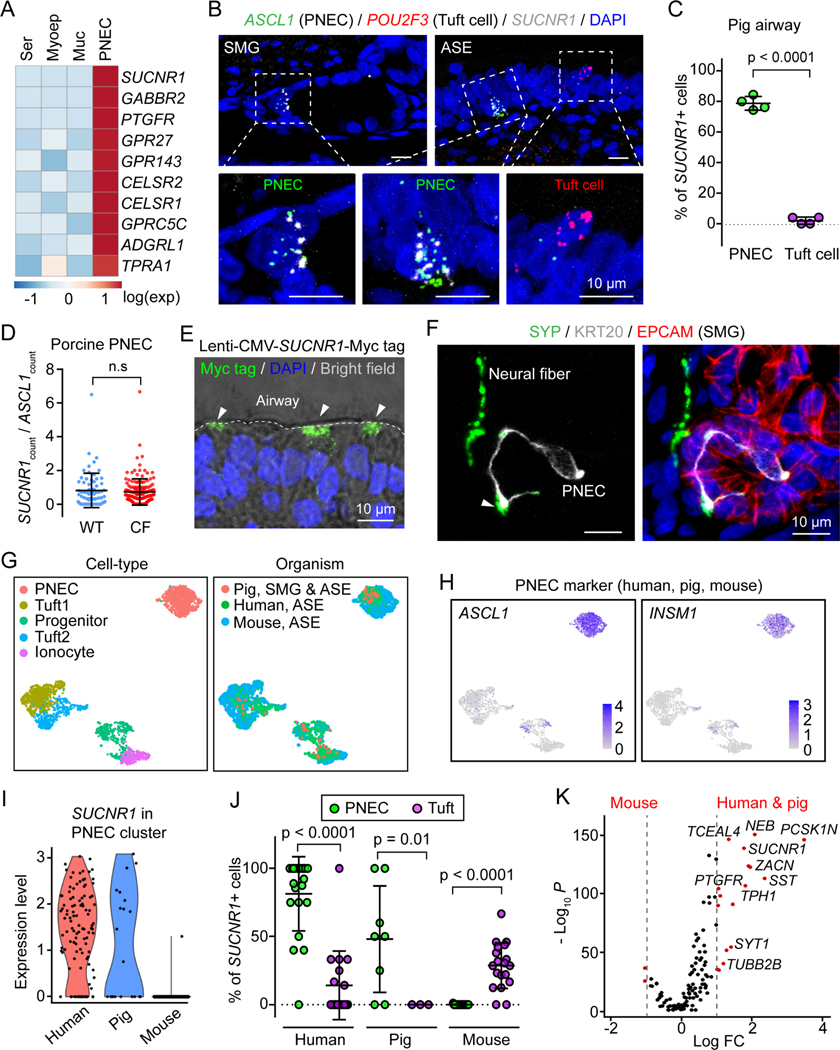
Porcine and human PNECs express *SUCNR1*. **A)** G protein-coupled receptors in SMG scRNA-seq data (Yu et al., 2022). Genes are ranked by false discovery rate (FDR) from the lowest to the highest. Ser, serous cell; Myoep, myoepithelial cell; Muc, mucous cell. See also Table S1. **B-D)** Characterization of *SUCNR1* expression using smFISH in porcine PNECs. **B** is representative smFISH images of *SUCNR1*, *ASCL1*, and *POU2F3* in SMGs and airway surface epithelia (ASE). **C** is the percentage of cells that are positive for SUCNR1 for PNECs (224 cells) and tuft cells (88 cells). n = 4 pigs (2WT, 2CF); p < 0.0001, paired Student’s *t* test. **D** is normalized *SUCNR1* level (smFISH counts) in WT and CF PNECs (224 cells). P value was calculated based on individual SMGs; p > 0.05 is considered as not significant (n.s), unpaired Student’s *t* test. **E)** Apical distribution of SUCNR1 in airway surface epithelia. Newborn pig trachea was infected with lentivirus carrying CMV promoter driven Myc-tagged *SUCNR1* cDNA. After one week of *ex vivo* culture, tissues were sectioned and stained with anti-Myc antibody. Dashed line highlights airway surface, arrowheads point to cells with apical Myc tag staining. **F)** Representative immunofluorescent images of neuron fibers (SYP+, green) and PNECs (KRT20+, gray) in porcine SMGs (EPCAM+, red). Arrowhead highlights the proximity of PNECs and neurons. **G)** Uniform manifold approximation and projection (UMAP) of pig, human, and mouse airway rare cell types grouped by cell-type (left) and organism (right). **H)** Expression of PNEC markers (*ASCL1*, *INSM1*) in UMAP of pig, human, and mouse airway rare cell types. **I)** Expression of *SUCNR1* in the PNEC cluster of human, pig, and mouse airway scRNA-seq data. **J)** Percentage of *SUCNR1*+ cells in PNECs and tuft cells (tuft1 plus tuft2) in human, pig, and mouse airway scRNA-seq datasets. Data points represent biological samples from the original scRNA-seq datasets. P values are shown in graph, unpaired Student’s *t* test. **K)** Comparison of PNEC markers between human and pig vs. mouse. Marker genes for both human and pig PNECs are shown. LogFC, fold change at natural log scale. See also Figure S3.

To investigate a potential chemosensory function for SMG PNECs, we focused on G protein-coupled receptors because of their critical role in many sensory systems and their prominence as therapeutic targets (Rosenbaum et al., 2009). We identified several G protein-coupled receptors that were enriched in SMG PNECs vs. other SMG cell types (Figure 2A). We focused on the succinate receptor 1 (SUCNR1) for three reasons. First, it was highly expressed and specifically enriched (lowest false discovery rate) in PNECs (Figure 2A; Table S1). Second, SUCNR1 is reported to be involved in chemosensation by rare epithelial cells in other organs (Schneider et al., 2018). Third, succinate is the ligand for SUCNR1, and succinate is abundant in the airway surface liquid of inflamed airways of humans and mice (He et al., 2004; Riquelme et al., 2020; Riquelme et al., 2019). For example, in mice infected with *Pseudomonas aeruginosa*, succinate levels in airway surface liquid were reported at ~1 mM in uninfected lungs and 10 mM in infected lungs, and in the sputum of CF patients, succinate concentrations were increased 10-fold (Riquelme *et al*., 2019). Using smFISH, we localized *SUCNR1* in SMG PNECs (*ASCL1*+) (Figure 2B). SMG PNECs and surface PNECs had similar labeling of transcripts, and *SUCNR1* was also present in PNECs located in the airway surface epithelium (Figures 2B and 2C). In addition, we compared *SUCNR1* expression between WT and CF PNECs and found no difference (Figure 2D). Previous studies indicate that SUCNR1 is localized to the apical membrane of epithelial cells in mouse kidney and intestine (Lei et al., 2018; Robben et al., 2009; Schneider *et al*., 2018); although we obtained four different commercially available antibodies, we were not able to obtain specific immunostaining of SUCNR1 in human or pig airway epithelial cells. Therefore, we expressed Myc tag-labeled SUCNR1 in airway epithelia and found that it localized to the apical membrane (Figure 2E). As expected for a chemosensory function, we also found SMG PNECs in proximity with neurons (Figure 2F).

In mouse airways, instead of PNECs, *SUCNR1* is expressed in tuft cells, another rare epithelial cell type involved in chemosensation (Montoro et al., 2018; Plasschaert et al., 2018). However, porcine SMGs lack tuft cells, and although porcine surface epithelia contain tuft cells, they lack *SUCNR1* (Figures 2B and 2C). To further investigate *SUCNR1* expression in rare cell types across species, we integrated our scRNA-seq data with scRNA-seq datasets from published human and mouse airways and then re-clustered cells into five cell types: PNEC, progenitors of these rare cell types, two subtypes of tuft cells, and ionocytes (Figures 2G, 2H, and S3) (Deprez et al., 2020; Goldfarbmuren et al., 2020; Montoro et al., 2018; Plasschaert et al., 2018). In humans and pigs, *SUCNR1* was predominantly expressed in PNECs, but in mice it was only in tuft cells (Figures 2I and 2J). We also found multiple additional genes that were enriched in human and pig PNECs but not in mouse PNECs (Figure 2K). Similar expression of chemosensory-related genes in human and pig PNECs suggest that they have conserved functions in these two organisms.

### Molecules on the airway surface diffuse into SMGs

**Figure 3 |:**
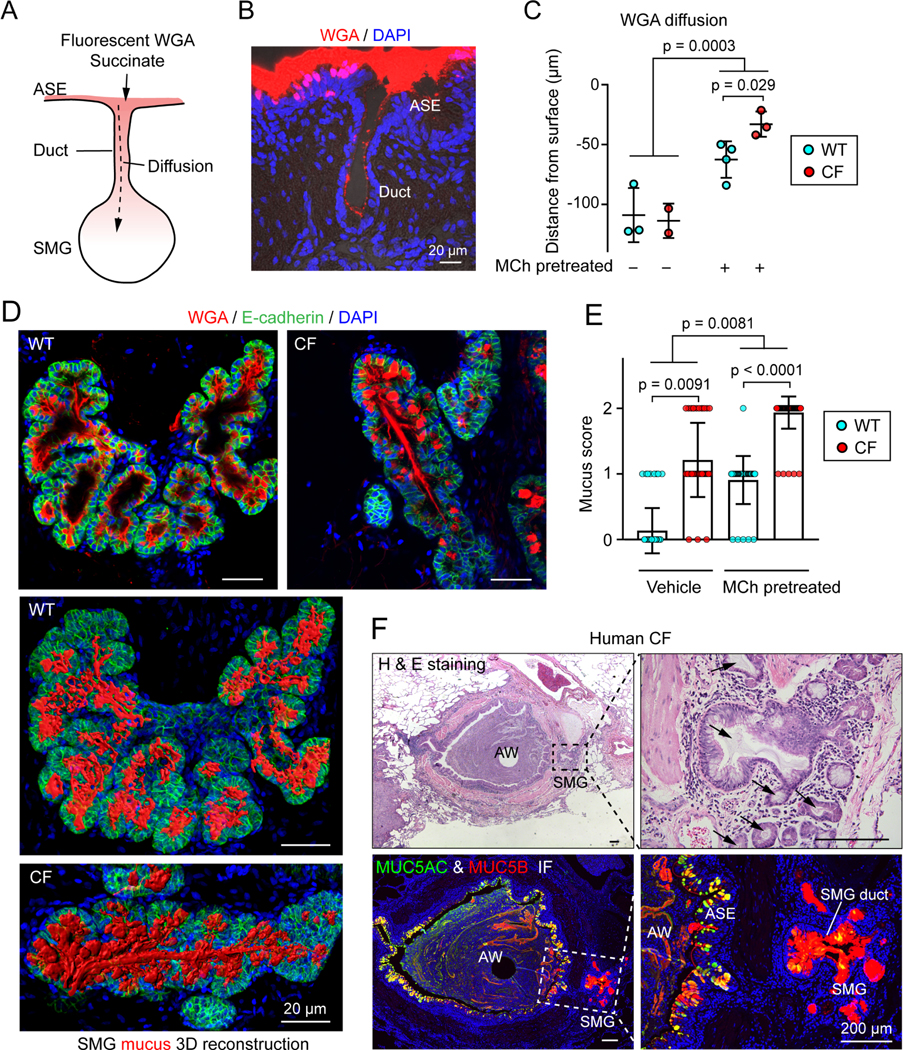
Airway surface molecules diffuse into SMGs through the SMG duct. **A)** Model of airway surface molecule diffusion in SMG lumen. **B)** Representative image of rhodamine-labeled wheat germ agglutinin (WGA) diffusion into SMGs. **C)** Summary of distance that rhodamine-labeled-WGA (0.028 mM) diffused down SMG lumen. Tracheal segments were pre-treated with or without 10 μM methacholine (to induce mucus secretion) and washed with Hank’s balanced salt solution 3 X 10 minutes before the diffusion assay. The diffusion distance was calculated based on the mean of 5 SMGs with the largest value from each pig. Each data point represents a pig; p values are shown in graph, Student’s *t* test. **D, E)** Quantification of mucus obstruction in SMG lumen. Panel **D** shows immunostaining of MUC5B mucin (WGA+, red) in the SMG acinus (E-cadherin+) of WT and CF pigs. Representative individual images (top panels) and three-dimensional SMG mucus models (lower two panels) are shown. Panel **E** is the summary of mucus obstruction score in the lumen of WT and CF SMGs. See Figure S4D for scoring method. Each data point represents a SMG, n = 3–5 pigs each group; p values are shown in graph, unpaired Student’s *t* test. **F)** Mucus obstruction in the airway and SMGs in lung from a person with CF. Left two images are hematoxylin and eosin (H & E) staining and immunostaining for MUC5AC (green) and MUC5B (red). Image shows an airway (AW) and one of the SMGs (in dashed box). Airway surface epithelia (ASE) has MUC5AC and MUC5B staining and SMG has MUC5B staining. Right two images show SMG with a lumen filled with MUC5B mucin (arrows). See also Figure S4.

If succinate in the airway lumen is to reach SUCNR1 on the apical surface of PNECs in SMGs, it must travel down the SMG duct (Figure 3A). We tested the ability of substances to diffuse down SMG ducts using rhodamine-labeled wheat germ agglutinin (WGA); we used WGA because it binds to cell membranes and mucin thereby avoiding washout during preparation of the sections. Although the labeled dye (36,000 g/mol) is much larger than succinate (118 g/mol), we readily detected it throughout the SMG lumen 10 min after application (Figures 3B, 3C, and S4A). We obtained similar data with CellMask (~700 g/mol), which binds to cell membranes (Figures S4B and S4C). Finding that material on the cell surface gains access to SMGs is consistent with previous reports that when replication incompetent viruses expressing a reporter are instilled into the trachea, they infect SMG cells, suggesting that virus travels down the duct and into the SMGs of surface molecules (Cao et al., 2013; Cooney et al., 2018; Griesenbach et al., 2011).

We predicted that mucus flowing up the SMG duct would reduce flow of surface molecules down the duct. Consistent with that expectation, stimulating mucus secretion with methacholine reduced penetration of labeled WGA (Figures 3C and S4A). In CF, mucus is abnormally elastic and viscous, has an increased protein concentration, and previous studies reported that it filled SMG ducts (Ostedgaard et al., 2017; Stoltz et al., 2015; Zuelzer and Newton, 1949). We further investigated whether mucus filled the acinar lumen of CF SMGs. After stimulating CF porcine SMG tissues with methacholine, we observed mucus obstructing the acinar lumen and duct (Figures 3D, 3E, and S4D; Video S1). We observed similar mucus obstruction in SMGs from people with CF (Figure 3F). These results suggested that mucus in CF SMGs might impair entry into the gland. Accordingly, methacholine stimulation decreased penetration of the WGA and CellMask tracers into CF glands (Figures 3C and S4A-S4C). These results suggest that mucus obstruction in CF may reduce or slow the movement of airway surface molecules into SMGs. Consequently, PNEC-derived chemosensation may be attenuated in CF SMGs.

### Succinate stimulates SUCNR1 on SMG PNECs to release ATP and facilitate SMG contraction

**Figure 4 |:**
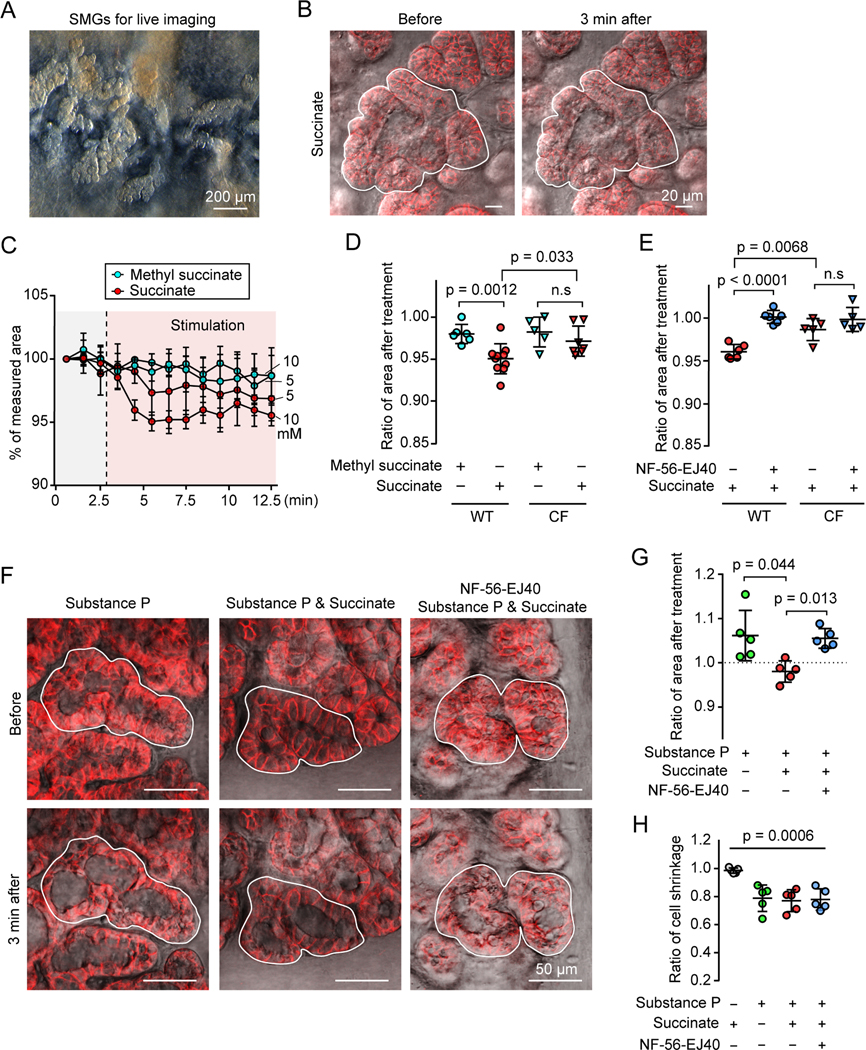
Extracellular succinate stimulates SUCNR1-dependent SMG contraction. **A)** Representative reflected light image of SMGs in newborn pig trachea after cartilage and smooth muscle layers were removed. **B)** Representative images of SMGs (EPCAM+, red) before and after succinate treatment. Closed lines outline the selected SMG regions for area measurement. **C)** Summary of SMG area treated with succinate or methyl succinate during live imaging of SMGs. 3 glands each group. **D, E)** Quantification of succinate-induced SMG contraction. Relative SMG area 3 minutes after a treatment. Panel **D,** SMGs were treated with 10 mM methyl succinate or succinate. Panel **E**, Airway surface epithelia were removed. SMGs were pre-incubated with or without SUCNR1 antagonist NF-56-EJ40 (5 μM) and induced with succinate. Data points are the mean of 3 SMGs from each pig; p values are shown in graph; p > 0.05 is considered as not significant (n.s), Student’s *t* test. **F, G**) Succinate prevents SMG swelling due to mucus secretion triggered by substance P. Panel **F**, representative images of SMGs that were pre-incubated with or without NF-56-EJ40 and treated with substance P and/or succinate. Panel **G**, quantification of SMG swelling response to substance P and/or succinate treatment. P values are shown in graph, n = 5 pigs, paired Student’s *t* test. **H**) Quantification of acinar cell shrinkage under succinate and/or substance P stimulation. P = 0.0006, n = 5 pigs, one way ANOVA. See also Figure S5.

To identify a potential physiological role for succinate-SUCNR1 signaling, we removed the smooth muscle and cartilage layers of newborn pig trachea so that we could visualize SMGs and assess their mucus secretion and contraction (Figure 4A) (Choi et al., 2009). Adding succinate to the airways did not induce observable mucus secretion as assessed by changes in cell volume (Lee and Foskett, 2014). However, succinate did induce SMG contraction, and contraction depended on the succinate concentration (Figures 4B-4D; Video S2). In contrast, methyl succinate, which does not stimulate SUCNR1 (He et al., 2004; Lei et al., 2018), failed to stimulate contraction.

To rule out the possibility that succinate-activated PNECs in surface epithelia produced the SMG contraction, we removed airway surface epithelia including surface PNECs by scraping (Yu et al., 2022). In the absence of surface epithelia, succinate stimulated SMG contraction and a *SUCNR1* specific antagonist NF-56-EJ40 abolished the effect (Haffke et al., 2019) (Figure 4E). We did not observe significant contraction in CF SMGs (Figures 4D and 4E), in which mucus obstruction may have attenuated or slowed the rate of succinate movement into the gland.

We also considered the possibility that succinate might activate other *SUCNR1*-expressing cell types to cause SMG contraction. A small percentage of myofibroblasts express *SUCNR1* (Figures S5A and S5B). However, myofibroblasts including *SUCNR1* positive myofibroblasts are localized beneath the SMGs rather than surrounding them (Figure S5C), and most of that tissue was removed before we assayed SMG contraction. Consistent with these observations, succinate did not induce myofibroblast or smooth muscle contraction (Figure S5D). These results indicated that SUCNR1 in SMG PNECs triggered succinate-induced SMG contraction.

The effect of succinate on SMGs contrasts with that of the neurotransmitter, substance P. Whereas succinate stimulates SMG contraction but not mucus secretion, substance P stimulates mucus secretion but not contraction (Choi *et al*., 2007; Choi *et al*., 2009; Khansaheb *et al*., 2011). Substance P stimulation causes release of apically located mucus-containing vesicles and rapid cell volume loss; as a result, the cells thin in the apical-basolateral plane, the gland lumen fills with mucus, and without contractile forces, the gland swells (Choi *et al*., 2009; Ianowski et al., 2008; Khansaheb *et al*., 2011). We hypothesized that succinate-induced contraction would counteract this expansion of SMGs. Applying substance P alone induced SMG swelling (Figures 4F and 4G). However, adding succinate together with substance P prevented swelling. In both cases, the acinar cells thinned, indicating mucus secretion (Figures 4F and 4H). The SUCNR1 antagonist NF-56-EJ40 prevented the effect of succinate. These results suggest that succinate-triggered SMG contraction complements substance P-induced mucus secretion to promote mucus ejection from SMGs.

**Figure 5 |:**
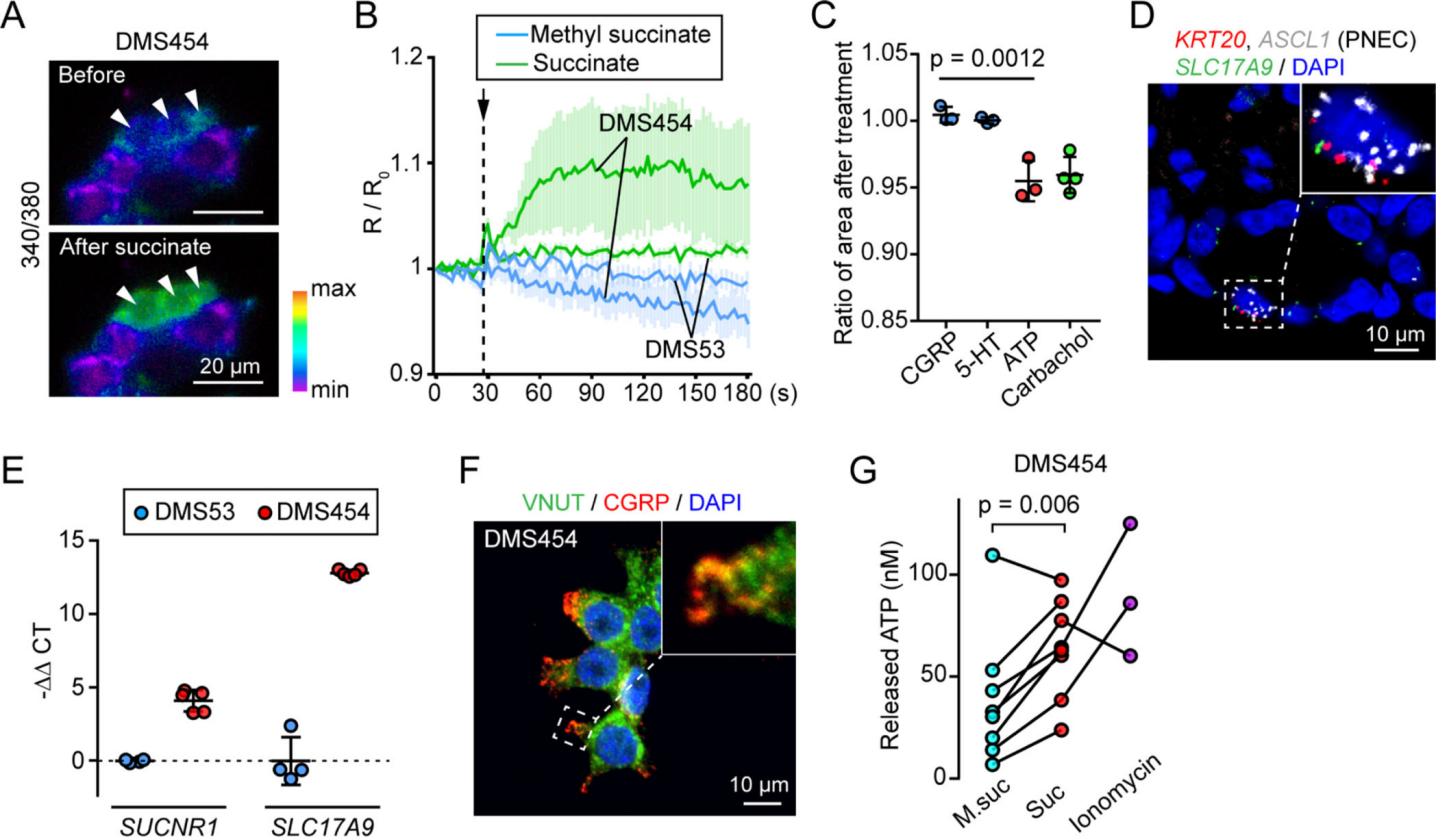
Succinate triggers ATP release from PNECs to elicit SMG contraction. **A)** Representative images of Fura-2 ratiometric Ca^2+^ imaging in DMS454 cells before and after succinate treatment. Arrowheads highlight the cell’s response to succinate. **B)** Summary of ratiometric Ca^2+^ imaging (R/R_0_, R = 340/380) in DMS454 and DMS53 cells that were treated with succinate or methyl succinate. Data are mean ± SEM. **C)** Relative SMG area 3 minutes after CGRP (0.5 μM), 5-HT (40 μM), ATP (1 mM), and carbachol (10 μM) treatment. Each data point is the mean of 3 SMGs from a pig; p = 0.0012, one-way ANOVA. **D)** Representative smFISH images of SMG PNECs (*KRT20* and *ASCL1*+) expressing *SLC17A9* (VNUT). **E)** qRT-PCR of *SUCNR1* and *SLC17A9* in DMS53 and DMS454 cells. **F)** Representative immunofluorescent images of VNUT (green) and CGRP (red) in DMS454 cells. Enlarged image highlights the colocalization of VNUT and CGRP. **G)** ATP release from DMS454 cells treated with methyl succinate (5 mM), succinate (5 mM), or ionomycin (2 μM). Each data point is the mean of replicates from an independent experiment. Lines connect the same experiment across treatment groups; p = 0.006, paired Student’s *t* test. See also Figure S6.

To investigate signaling downstream of succinate-SUCNR1, we studied two PNEC-like small cell lung cancer cell lines, DMS454 and DMS53. These cell lines have gene signatures of PNECs including transcription factors (*ASCL1*, *INSM1*), neurohumoral genes (*CALCA*, *GRP*, *SST, CHGA, CHGB, DDC, TPH1*), and transporters (*CACNA1A, SCN9A, SLC38A11, ZACN*) (Figure S6). Importantly for our purposes, one of the cells has high *SUCNR1* expression, DMS454, and the other has very low *SUCNR1* expression, DMS53. Previous reports showed that activating *SUCNR1* increases the intracellular Ca^2+^ concentration, [Ca^2+^]_c_ (He et al., 2004; Robben et al., 2009; Wang et al., 2019). Consistent with that earlier work, we applied succinate and found that [Ca^2+^]_c_ increased in DMS454 cells but not in DMS53 cells (Figures 5A and 5B).

Previous studies have shown that increasing [Ca^2+^]_c_ elicits exocytosis of PNEC vesicles containing neurohumoral agents, including CGRP, 5-HT, and ATP (Garg et al., 2019; Nadjsombati et al., 2018; Noguchi et al., 2020). To determine which of these neurotransmitters were responsible for succinate-induced contraction, we applied them individually to SMGs and found that only ATP induced SMG contraction. The contraction was similar to that produced by carbachol used as a positive control (Figure 5C). These results suggested that ATP is the downstream signal of the PNEC succinate-SUCNR1 axis that induces SMG contraction.

Supporting that conclusion, in pig SMG PNECs and in human PNEC-like DMS454 cells, we found expression of transcripts for *SLC17A9* (Figures 5D and 5E), the gene encoding vesicular nucleotide transporter (VNUT), which packs ATP into neurovesicles (Estevez-Herrera et al., 2016; Masuda et al., 2016). We further confirmed that CGRP+ neurovesicles were positive for VNUT (Figure 5F). In addition, adding succinate to DMS454 cells released ATP (Figure 5G). As a positive control, we applied ionomycin, which increases [Ca^2+^]_c_ and also observed ATP release. Together, these results indicate that succinate activates SUCNR1 thereby increasing [Ca^2+^]_c_, eliciting ATP release, and stimulating SMG contraction.

### ATP stimulates myoepithelial cell P2Y1 receptors to facilitate SMG contraction

**Figure 6 |:**
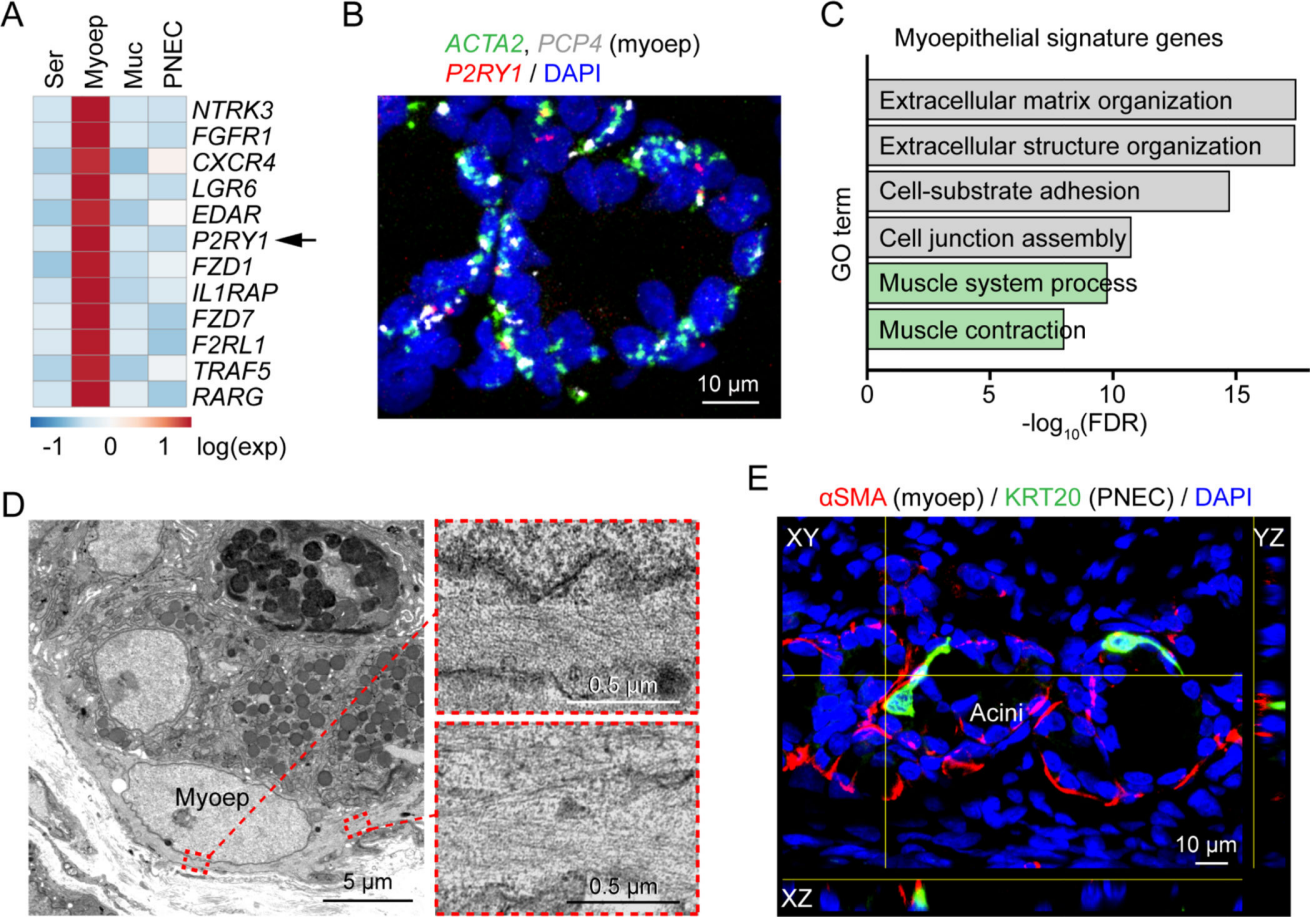
SMG myoepithelial cells express the purinergic receptor *P2RY1*. **A)** Heatmap of myoepithelial signature receptors in SMG scRNA-seq data (Yu et al., 2022). Genes are ranked by false discovery rate from the lowest to the highest. Ser, serous cell; Myoep, myoepithelial cell; Muc, mucous cell. **B)** Representative smFISH image of *ACTA2*, *P2RY1*, and *PCP4* in SMGs. Myoep, myoepithelial cell. **C)** Gene ontology (GO) analysis of myoepithelial-cell signature genes. Muscle contraction related pathways are highlighted in green. FDR, false discovery rate. **D)** Representative transmission electron microscopy images of myoepithelial cells in SMGs. Enlarged images show microfilaments of the myoepithelial cell. **E)** Representative image of PNEC (green) and myoepithelial cell (myoep, red) proximity in SMGs.

To identify a target cell-type for the ATP signal, we checked for purinergic gene expression in the scRNA-seq dataset from porcine SMGs (Yu et al., 2022). We found that *P2RY1*, a G protein-coupled receptor for extracellular ATP, was specifically expressed in myoepithelial cells (Figures 6A, 6B, and S7A; Table S2). Myoepithelial cells were a promising candidate to mediate the contractile activity of ATP. First, they are a contractile cell. Consistent with that function, SMG myoepithelial cells were enriched for expression of smooth muscle contraction-related genes, and electron microscopy showed abundant microfilaments (Figures 6C and 6D) (Haaksma et al., 2011; Horowitz et al., 1996; Shimura et al., 1986). Second, extracellular ATP is rapidly degraded by extracellular nucleotidases and thus only diffuses a short distance (Yegutkin, 2008; Zimmermann et al., 2012). Of note, myoepithelial cells wrap around the SMG acinus and thus PNECs would release ATP in close vicinity to myoepithelial cells (Figure 6E).

**Figure 7 |:**
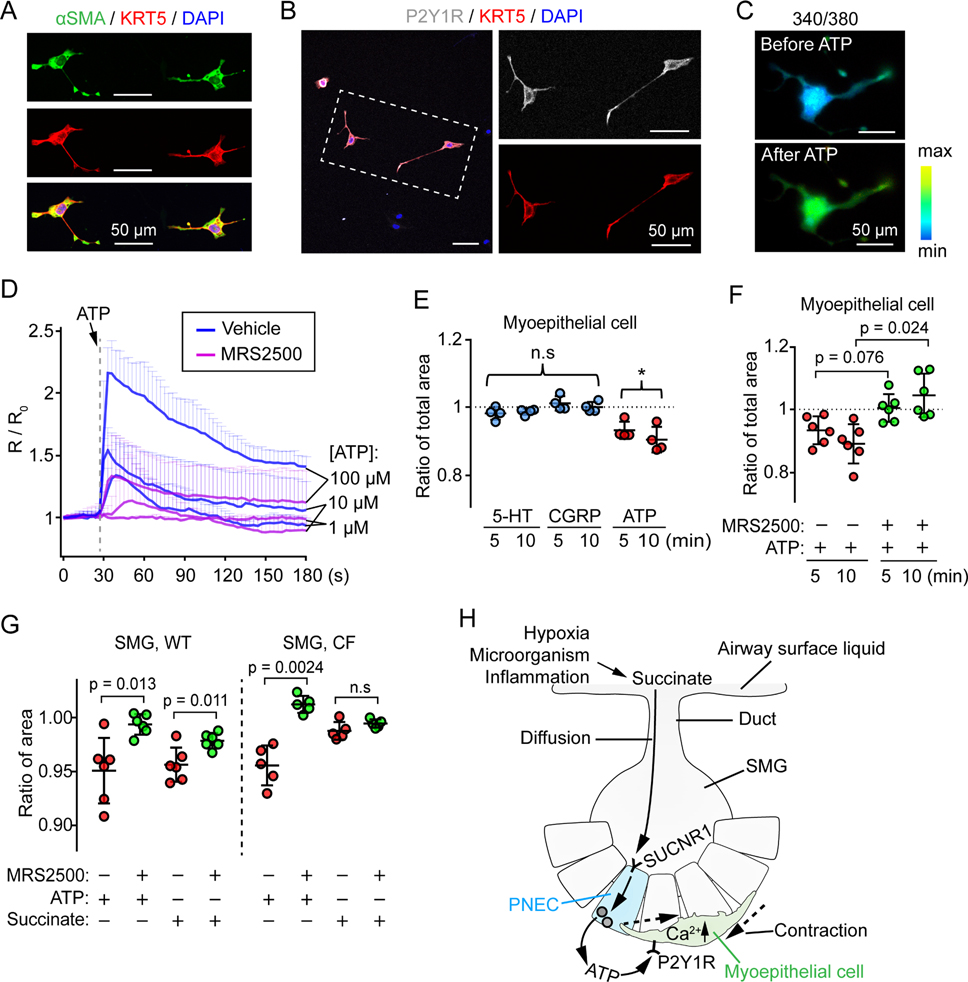
ATP-P2Y1R signaling controls myoepithelial cell contraction in SMGs. **A)** Characterization of cultured primary myoepithelial cells after 5 days of culture. aSMA and KRT5 are markers for myoepithelial cells. **B)** Representative images of primary myoepithelial cells (KRT5+, red) stained with P2Y1 receptor antibody (gray). **C)** Representative Fura-2 ratiometric Ca^2+^ images of primary myoepithelial cells before and after ATP. **D)** Summary of ratiometric Ca^2+^ imaging (R/R_0_, R = 340/380) in primary myoepithelial cells. Cells were pre-incubated with or without P2Y1 receptor antagonist MRS2500 (5 μM). Dashed line indicates the time when ATP (1, 10, 100 μM) was added. n = 3 pigs. **E, F)** ATP stimulates primary myoepithelial cell contraction (presented as ratio of total area after treatment). Panel **E**, effects of 5-HT, CGRP, or ATP on primary myoepithelial cell contraction. Panel **F**, P2Y1 receptor antagonist MRS2500 abolished ATP controlled primary myoepithelial cell contraction. Each data point represents the mean of imaged cells from a pig; p values are shown in graph; n.s, not significant; *, p < 0.05; one sample t test for panel **E**, paired Student’s *t* test for panel **F**. **G)** WT and CF SMG contraction with ATP (1 mM) or succinate (10 mM) treatment *ex vivo*. Each data point is the mean of 3 SMGs from an individual pig. P values are shown in graph, paired Student’s *t* test. **H)** Model of SMG PNEC sensing succinate to trigger myoepithelial contraction. PNECs located in airway SMGs express SUCNR1, the receptor for succinate. Under hypoxia, infection, and inflammation, succinate is generated by macrophages, other epithelial cells, or microorganisms in airway surface liquid, which diffuses into the SMGs. SUCNR1 on SMG PNEC responds to extracellular succinate by releasing ATP to the basolateral environment. ATP activates P2Y1 receptors on myoepithelial cells to cause Ca^2+^-dependent myoepithelial contraction. See also Figure S7.

We tested the response to ATP in primary cultures of myoepithelial cells isolated from pig SMGs (Figures 7A and 7B). Applying ATP increased [Ca^2+^]_c_ in myoepithelial cells, and the P2Y1 receptor specific antagonist MRS2500 prevented the rise in [Ca^2+^]_c_ (Figures 7C, 7D, and S7B; Video S3). ATP caused myoepithelial cell contraction, whereas 5-HT and CGRP did not (Figure 7E). Of note, the scRNA-seg data show that myoepithelial cells express little or no genes encoding 5-HT or CGRP receptors (Figure S7C). MRS2500 blocked myoepithelial cell contraction in response to ATP application (Figures 7F and S7D; Video S4). These results are consistent with studies showing that ATP induced myoepithelial cell contraction in contractile mammary and lacrimal glands (Garcia-Posadas et al., 2020; Nakano et al., 1997). Last, the P2Y1 receptor inhibitor MRS2500 also blocked both succinate- and ATP-induced contraction of SMGs (Figure 7G), suggesting that PNEC-myoepithelial cell interactions were responsible for succinate-induced SMG contraction.

## DISCUSSION

These findings reveal that PNECs within SMGs orchestrate a local circuit, detecting succinate from the airway surface and releasing ATP that stimulates myoepithelial cells to contract SMGs. This chemosensory:effector system is enabled by the cellular structure of PNECs and SMGs (Figure 7H). PNECs reach the apical surface where they can sense succinate that diffuses down the SMG duct. Their long basolateral extensions allow them to reach a large area and exert an outsized signaling role that belies their rare occurrence. Myoepithelial cells wrap tightly around the glands, placing them in close proximity to PNECs. Thus, when PNECs release ATP, myoepithelial are positioned to detect and respond to a signal with a short lifetime and limited range (Yegutkin, 2008; Zimmermann et al., 2012).

Succinate is a citric acid cycle metabolite and an important extracellular signal (Hochachka and Dressendorfer, 1976; Mills and O’Neill, 2014; Nunns et al., 2020). Hypoxia and ischemia cause an imbalance in cellular energy production, accumulation of succinate, and its release from cells to signal the presence of local stress (Chouchani et al., 2014; Tannahill et al., 2013). Succinate is also a key indicator and signal of inflammation, and activated macrophages produce succinate in response to inflammatory signals (Littlewood-Evans et al., 2016; Tannahill et al., 2013). For example, the profuse inflammation that characterizes cystic fibrosis lungs infected with *Pseudomonas aeruginosa* increases airway epithelial succinate production and markedly raises the succinate concentration in airway surface liquid (Riquelme et al., 2019). Microorganisms also produce succinate that can stimulate SUCNR1 (Fischbach and Sonnenburg, 2011; Gilroy et al., 1988; Schneider et al., 2018). A key role for SUCNR1 has also been shown in mouse small intestine where SUCNR1 on the apical membrane of tuft cells senses extracellular succinate to initiate innate lymphoid type 2 cell related inflammation (Lei et al., 2018; Schneider et al., 2018). Thus, a variety of challenges increase local levels of succinate, which is sensed by SUCNR1 on PNECs.

In addition to local control, proximity between PNEC projections and neurons laying near the basal side of SMGs suggests that interactions between the two could also signal SMG contraction and/or secretion through neurohumoral control (Widdicombe and Wine, 2015). Based on previous work, we also speculate that SMG PNECs might initiate Type 2 inflammation as identified for allergen stimulation (Sui et al., 2018) and/or initiate repair mechanisms when hypoxia increases succinate levels (Shivaraju et al., 2021). Thus, SMG PNECs may direct respiratory host defense in more than one way.

Our results revealed some differences between PNECs in humans and pigs and those in mice. For example, human and pig PNECs express G protein-coupled receptor (*SUCNR1, PTGFR*), ion channel (*ZACN*), neuropeptide (*SST*) and cytoskeletal (*NEB*) genes that are not expressed in mouse PNECs. In addition, the cell types that express SUCNR1 diverge by species: PNECs in pigs and humans and tuft cells in mice. Although the reasons for these species’ differences are unknown, the obvious size divergence of these species may be an important contributing factor. Compared to the very narrow airways of mice, the large diameter airways of pigs and humans are more accessible to inhaled and aspirated particles. Thus, environmental challenges will differ and perhaps be greater and more varied in humans and pigs than in mice. Species differences in the genes for receptors and other proteins that PNECs express may allow large mammals to more effectively sense and respond to the environmental challenges they face. Distributing chemosensory receptors to PNECs rather than to tuft cells also enables signal amplification through the neurons that extensively innervate PNECs.

Discharge of mucus onto the airway surface entails regulatory control, release of mucus from SMG cells, and ejection of mucus from the glands into the airway lumen. Previous studies showed that substance P and vasoactive intestinal peptide (VIP) stimulate SMG cells to release mucus (Choi *et al*., 2007; Choi *et al*., 2009; Khansaheb *et al*., 2011). However, these neurotransmitters do not induce SMG contraction, and when the secreted mucus expands in the gland lumen, the gland swells. Our findings indicate that succinate has a complementary action, stimulating contraction of the myoepithelial cells that wrap closely around the glands. Thus, succinate-triggered gland contraction would prevent swelling and promote mucus ejection into the airway lumen. This regulatory process may be especially important in inflammation and infection. Inflammatory diseases such as chronic obstructive pulmonary disease increase SMG innervation with nerve terminals containing substance P and VIP (Lucchini et al., 1997; Vatrella et al., 2010). Inflammation can also markedly increase succinate levels at the airway surface (Littlewood-Evans *et al*., 2016; Tannahill *et al*., 2013). With inflammation, succinate on the airway surface would diffuse down into SMGs to reach its receptor on the apical membrane of PNECs, and substance P and VIP released from adjacent nerve terminals would reach their receptors on the basolateral membrane of gland cells. This dual regulatory system with sensors on both sides of the epithelium may provide the greatest control over SMG function. We speculate that this organization may ensure efficacy of a process that is required to kill microbes and clear the airways when the lung is challenged by inflammation and infection.

Finding that PNECs sense and initiate a local SMG response to the inflammatory molecule succinate indicates that PNECs make an important contribution to respiratory host defense. However, PNECs may also contribute to disease. In murine models of asthma, PNECs amplify goblet cell hyperplasia (Sui et al., 2018). In humans, PNECs are the cell type of origin for small cell lung cancer (Ouadah et al., 2019). In a murine model of neuroendocrine hyperplasia of infancy (NEHI) with increased numbers of PNECs, elevated CGRP levels increase endothelial permeability leading to increased lung liquid and hypoxemia (Xu et al., 2022). An increased number of PNECs has also been reported in a variety of lung diseases, although whether that is protective or detrimental remains uncertain (Noguchi et al., 2020; Xu et al., 2020). Our results suggest that CF may at least partially compromise respiratory host defense when abnormally elastic and viscous mucus impairs penetration of succinate into SMGs and PNECs. Consequently, contraction to succinate may be delayed or attenuated in CF SMGs. This defect might also apply to other airway surface molecules and pathogens in CF, as both PNECs and secretory cells in SMG have many other chemosensory receptors (Yu et al., 2022). Thus, we speculate that impaired access to SMG PNECs may further disrupt airway defenses in CF potentially worsening CF lung infection.

### Limitations of the Study

This study has advantages and limitations, and both revolve around use of pigs rather mice, a more commonly studied species. An obvious advantage of using pigs is that their lungs more closely resemble those of humans than do mouse lungs, and like humans, pig airways have abundant SMGs whereas mouse intrapulmonary airways have only a few SMGs that are located near the larynx (Meyerholz et al., 2018; Tata et al., 2018; Widdicombe et al., 2001). An obvious limitation of using pigs is that they lack the extensive genomic tools available to manipulate the mouse genome. Another factor that is both an advantage and a limitation is that we primarily studied very young animals, although we did limited studies in a few older animals and human lungs. The advantage of studying young pigs is that their lungs will not be altered by infection, inflammation, or environmental factors. The limitation is that those challenges later in life might alter PNEC structure and function. Our current work can provide a baseline for future research in older animals with disease and/or with various challenges.

## STAR METHODS

### RESOURCE AVAILABILITY

#### Lead contact

Further information and requests for resources and reagents should be directed to and will be fulfilled by the Lead Contact, Michael J. Welsh (michael-welsh@uiowa.edu).

#### Materials Availability

This study did not generate unique reagents.

#### Data and Code Availability

Single-cell RNA sequencing data reported in this paper have been published (Yu et al., 2022) and deposited in the Gene Expression Omnibus (GEO) database, https://www.ncbi.nlm.nih.gov/geo (accession no. GSE185849).

No custom codes were developed in this study.

### EXPERIMENTAL MODEL AND SUBJECT DETAILS

#### Human donor lungs and primary airway epithelial cells

Human lung tissues were obtained, with informed consent of the donors, from the Iowa Donor Network through the In Vitro Models and Cell Culture Core of the University of Iowa. Human primary airway epithelial cells were prepared based on a previous protocol (Karp et al., 2002) by the In Vitro Models and Cell Culture Core of the University of Iowa. All studies were approved by the University of Iowa Institutional Review Board.

#### Pigs and tissue harvest

We obtained pigs from Exemplar Genetics. *CFTR*^−/−^ pigs were generated as previously described (Rogers et al., 2008b). Newborn pigs (8–15 hours after birth) and 1–2 months old pigs were delivered to the University of Iowa, maintained in accordance with the University of Iowa Animal Care and Use Committee guidelines, and anesthetized and euthanized the same day. Tracheal segments were harvested and kept in cold Krebs bicarbonate buffer before further procedure. All studies were reviewed and approved by the Animal Care and Use Committee of the University of Iowa.

### METHOD DETAILS

#### Cell culture

DMS454 (Sigma, 95062832–1VL, origin from the Public Health England) and DMS53 (ATCC, CRL2062) cells were cultured in the Waymouth′s medium with 2 mM glutamine and 10% fetal bovine serum. We incubated flasks or 24-well plates (Cellvis, P24–1.5P) with 3–5% collagen (Stemcells Technologies, 04902) for 1–2 hours before DMS454 culture. After 3–5 days of culture, cells were subjected to immunostaining, RNA extraction, or live imaging.

For primary myoepithelial cell culture, tracheal SMGs from 1 to 2 months old pigs were dissected and dissociated as previously described (Yu et al., 2022). SMG single cells were cultured in small airway epithelial cell growth medium with A83–01 (1 μM, Stemcells Technologies, 72022), CHIR 99021 (1 μM, Stemcells Technologies, 72052), DMH-1 (1 μM, Stemcells Technologies, 73632), Y-27632 (10 μM, Stemcells Technologies, 72302), gentamycin (1/1000, IBI Scientific, IB02030), and penicillin/streptomycin (1/500, Life Technologies, 15140122) on 0.5% Matrigel (Fisher scientific, CB-40230A) in glass bottom 24-well plates (Cellvis, P24–1.5P) (Tata et al., 2018). Cultured primary myoepithelial cells were confirmed by immunostaining of KRT5 and αSMA after 5 days of culture. Live imaging (Ca^2+^ imaging or contraction imaging) was conducted at day 4 of culture.

#### Immunostaining

Formalin-fixed paraffin-embedded sections, cryosections, and whole tracheal epithelial tissues were prepared as previously described (Yu et al., 2022). Sections and tissues were sequentially permeabilized with 0.2% Triton X-100 in 1X DPBS, blocked with 10% serum, incubated with primary antibodies and secondary antibodies. After that, sections and tissues were mounted in antifade mounting medium with 4′,6-diamidino-2-phenylin-dole (DAPI), kept in 4 °C overnight, and imaged with confocal microscopy. The details of the antibodies were listed in Table S3.

For exogenous Myc-tagged SUCNR1 expression and labeling, pieces of newborn pig trachea (~6*6mm) were apically infected with 50 μl *SUCNR1*-Myc tag lentiviral particles (1*10^7^ TU/mL) (OriGene, RC205888L1V) in Dulbecco’s Modified Eagle’s medium with 0.1% lysophosphatidylcholine for four hours. Explants were washed with 1X DPBS, cultured at the air-liquid interface for one week. Explants were washed, fixed, sectioned, and immunostained with anti-Myc antibody.

We used Imaris software from Bitplane for mucus three-dimensional reconstruction in whole SMGs stained with WGA and E-cadherin. A ‘surface’ was added to the WGA channel to reconstruct MUC5B mucin in mucous cells and mucus in SMG lumen.

#### Single-molecule fluorescence *in situ* hybridization (smFISH)

An smFISH method named proximity ligation *in situ* hybridization was performed based on a published protocol (Nagendran et al., 2018). We followed the typical protocol with some modifications as previously described (Yu et al., 2022). In brief, tracheal segments were fixed in 4% formaldehyde at 4 °C for 24 hours. Cryosections (10 μm) were cut, post-fixed in cold 4% formaldehyde, incubated in citrate buffer (Thermo Fisher Scientific, 005000) at 65 °C, and digested in pepsin solution. Next, tissue sections were sequentially incubated with short-paired hybridization probes, circle & bridge probes, T4 DNA ligase solution (ligation), DNA polymerase solution (rolling circle amplification), and fluorophore conjugated labeling probes. Last, sections were mounted in anti-fade mounting medium with DAPI and imaged with confocal microscopy. The short mRNA sequences that were targeted by the paired hybridization probes were listed in Table S3.

#### Transmission electron microscopic imaging

Tracheal segments less than 1 mm^3^ from newborn pigs were cut, rinsed in cold 1X DPBS, and fixed in sodium cacodylate solution with 2.5% glutaraldehyde. Tissues were post-fixed in 2% osmium tetroxide and en bloc stained with 2.5% uranyl acetate. Next, tissues were dehydrated, infiltrated with Eponate 12, and polymerized at 60 °C for one day. 70 nm sections were cut and counterstained with 5% uranyl acetate and Reynold’s lead citrate. Images were taken using a transmission electron microscope (JEOL, JEM1230) with a Gatan UltraScan 2k x 2k CCD camera.

#### Analysis of available scRNA-seq datasets

Our SMG single-cell RNA sequencing (scRNA-seq) data were previously published (GSE185849) (Yu et al., 2022). PNEC and myoepithelial cell marker gene sets (Table S1 and S2) were subjected to receptor, ion transporter, and gene set enrichment analysis. To simplify the visualization of the data, we only included epithelial cell types from SMG acinus in heatmap results.

We also pulled out SMG PNEC cells and rare epithelial cell types (PNEC, tuft cell, and ionocyte) from our published large and small airway scRNA-seq data (GSE150211) (Thurman et al., 2022) for rare cell type integration analysis in pigs, humans, and mice. Droplet-based scRNA-seq datasets of human and mouse airways were download from the following links: Deprez dataset (Deprez et al., 2020) in human: https://www.genomique.eu/cellbrowser/HCA/; Goldfarbmuren dataset (Goldfarbmuren et al., 2020) in human: GSE134174 (https://www.ncbi.nlm.nih.gov/geo/query/acc.cgi?acc=GSE134174); Montoro datasets (Montoro et al., 2018) in mouse: GSE103354 (https://www.ncbi.nlm.nih.gov/geo/query/acc.cgi?acc=GSE103354), including “PulseSeq” and “Trachea droplet”; Plasschaert dataset (Plasschaert et al., 2018) in mouse: GSE102580 (https://www.ncbi.nlm.nih.gov/geo/query/acc.cgi?acc=GSE102580).

Pig and mouse gene symbols were converted to human orthologous genes before analysis. All datasets were integrated together using the Seurat R toolkit (Stuart et al., 2019). Cells were clustered and visualized in two-dimensional uniform manifold approximation and projection (UMAP) (Becht et al., 2018).

#### Pathway enrichment analysis

Pathway enrichment analysis was performed using the “enrichGO” of the clusterProfiler 3.14 R package (https://guangchuangyu.github.io/software/clusterProfiler/) (Yu et al., 2012). PNEC marker genes (Table S1) and myoepithelial cell marker genes (Table S2) were converted to human orthologs and subjected for “enrichGO” analysis using human gene ontology database as the reference.

#### Fluorescent dye diffusion in SMG ducts

WT and CF newborn pig tracheal segments were washed in warm (37 °C) Hank’s balanced salt solution (HBSS) and incubated with or without 10 μM acetyl-β-methylcholine (methacholine) for 10 minutes in HBSS at 37 °C. Tracheal segments were washed with HBSS at 37 °C for three times, 10 minutes each time. Next, tissues were incubated in HBSS with rhodamine-labeled WGA (28 μM) or CellMask (50 μM, Thermo Scientific, C37608) for 10 minutes at 37 °C. Tissues were washed with warm HBSS and fixed in 10% neutral buffered formalin for 2 hours at room temperature. Tissues were dehydrated in 30% sucrose, embedded in OCT compound, and cut into 10 μm sections. 12 serial sections were collected, washed, mounted, and imaged by confocal microscopy. We took a single layer image for every SMG that had an obvious duct in sections. The distance between the furthest fluorescent dye in SMGs and airway surface basal layer was calculated in ImageJ. Only the largest value from the serial sections of an individual SMG was included for analysis.

#### SMG live imaging *ex vivo*

Newborn pig tracheal epithelia with SMGs were peeled off from the cartilage and smooth muscle layers. Epithelial tissues were washed with Krebs bicarbonate buffer and stained with fluorescent-labeled EPCAM antibody (Thermo Fisher Scientific, 12–5791-82) for 1 hour at room temperature on a shaker. Tissues were washed, cut into ~2*10 mm pieces, and moved to glass bottom dishes (3 cm diameter) with 2 ml Krebs bicarbonate buffer with the gland side facing down. A slice anchor (Warner instruments, 64–1419) was put onto the tissue to anchor the tissue to the bottom of the dish. Anchored tissues were moved to a live imaging system (Zeiss 880, 37 °C, 5% CO_2_). Submerged tissues were incubated with or without SUCNR1 antagonist NF-56-EJ40 (MedChem Express, HY-130246) and P2Y1R antagonist MRS2500 (Tocris, 2159) for ~30 minutes, respectively. Individual SMGs were imaged every 30 seconds for 16 or 26 cycles. Succinate (pH 7.4, Sigma, 224731), methyl succinate (pH 7.4, Millipore, M81101), ATP (1 mM, Sigma, A6419), substance P (2 μM, Sigma, 05–23-0600), serotonin (40 μM, Sigma, 14927), or CGRP (0.5 μM, GenScript, custom porcine CGRP) were added after the sixth or eighth imaging cycle.

For the SMG contraction analysis, SMGs highlighted with red fluorescence (EPCAM+) and high contrast in bright field were outlined. The area of the outlined region was measured in the Adobe Illustrator using the code from https://gist.github.com/bryanbuchanan/11387501. SMG contraction was determined by comparing the SMG area before and 3 minutes after treatments.

#### Extracellular ATP measurement

Equal amounts of DMS454 cells were seeded on collagen-treated 6-well plates. After 4 to 6 days of culture (60–80% confluence), cells were gently washed with warm HBSS. Then 200–300 μl warm HBSS containing succinate (5 mM), methyl succinate (5 mM), or ionomycin (2 μM) were gently added to each well. Plates were moved back to cell culture incubator. After 5 minutes, 100 μl supernatants from each well were collected for ATP measurement. ATP concentration was measured using a bioluminescence-based ATP Determination Kit (Thermo Fisher Scientific, A22066). ATP measurements were repeated in eight 6-well plate cultures on different days. We excluded individual wells that had large dry surface area (4 out of 48) during the 5-minute incubation period.

#### RNA extraction and qRT-PCR

Primary human airway epithelial cells or small cell lung cancer cells were washed with cold 1X DPBS and lysed with 1 ml TRIzol (Invitrogen) for 5–10 minutes on a shaker at room temperature. RNAs were purified using a RNeasy kit (Qiagen, 58730).

cDNA libraries were prepared using the High-Capacity cDNA Reverse Transcription Kits (# 4368814, Thermo Fisher Scientific). 1 μg RNAs from each sample were added to 20 μl reaction mix with reverse transcriptase, random primers, and dNTPs. cDNA libraries were generated following typical thermal cycling condition from the kit.

Quantitative PCR was performed using the Fast SYBR Green Master Mix (Applied Biosystems-Life Technologies, 4385612). CT values of tested genes were normalized to the CT values of reference genes (*RPL13A* or *ACTB*) to generate ΔCT values. Fold changes were determined using ΔΔCT values. Primers used in qRT-PCR were listed in Table S3.

#### Primary myoepithelial cell contraction analysis

After 4 days of culture, primary myoepithelial cells were moved to a Zeiss 880 live imaging system. Cells were pre-incubated with MRS2500 for at least 30 minutes. Myoepithelial cells were determined based on their angular-shaped cell body and multiple elongated branches in brightfield. Images were taken every 1 or 5 minutes for 10 minutes total before and after 5-HT, CGRP, or ATP treatment.

To quantify myoepithelial cell contraction, we sequentially connected the tips of all branches of a myoepithelial cell with linear lines to generate an area that included the entire cell. We defined that area as total area (TA). The TA of myoepithelial cells at different time points was calculated as TA_time t_/TA_time_=0.

#### Intracellular calcium imaging

Cells were washed with warm HBSS for 3 times. The relative changes of intracellular Ca^2+^ concentration was measured using either Fura-2 (Thermo scientific, F1221) or BioTracker 609 Red Ca2+ AM Dye (Sigma, # SCT021) following manufacturer’s protocols. During the imaging process, cells were either supported with a 37 ^o^C heat stage or maintained in humidified 37 °C, 5% CO_2_ environment.

For Fura-2 based ratiometric Ca^2+^ measurement, images were taken every 2 or 3 seconds using an EM-CCD camera (Evolve 512, Photometrics, USA) on an inverted microscope (Eclipse TE200, Nikon, USA). 340 nm and 380 nm excitation fluorescence images over a 3-minute period were taken. Ratiometric 340/380 images were generated, and fluorescence density values were exported using the NIS-Elements AR 4.6 software.

For BioTracker based fluorescence density measurement, images were taken every 30 seconds using a Zeiss 880 confocal microscope with the maximum pinhole. Images were aligned using the “Template matching” plugin in ImageJ to minimalize horizon shift (Tseng et al., 2011). Background fluorescence was filtered using the function “Subtract background” in ImageJ. A region of interest was selected for each cell, and the fluorescence density of the selected region was measured in ImageJ.

#### Tracheal segment contraction analysis

Trachea rings were mounted in the Radnoti Tissue Bath Systems. After mounting, the tissues were allowed to equilibrate at tension for an hour before treatment. A serial concentration of succinate (0.1, 1, 10, 50 mM) and carbachol (0.1 mM) were sequentially added into the bath system. The airway smooth muscle contraction force was determined by obtaining the maximal values of each condition. The measurement was conducted in a total of 8 trachea rings from 2 pigs.

#### Confocal imaging

Fluorescent images were taken using the Olympus FV3000 system. Whole mounted SMG tissues or tissue sections were scanned (average setting: line, 2 times) using either 20X or 63X lens with optical setting. Z-stack images were processed to either single z-stack images or z-stack videos.

Live images were taken using the Zeiss 880 system. 10X or 20X objectives were used for imaging. The pinhole of the scope was opened to maximum. Transmitted light channel and fluorescent channels were used to take brightfield and fluorescent images.

### QUANTIFICATION AND STATISTICAL ANALYSIS

#### Quantification of total cells and PNECs in individual SMGs

Whole newborn pig tracheal epithelia without cartilage and smooth muscle layers were fixed, permeabilized, and immunostained with epithelial cell marker E-cadherin and/or PNEC marker KRT20. For SMG total cell quantification, individual SMGs were imaged (20X lens, 1.05 μm per step, Olympus FV3000). SMG nuclei (E-cadherin positive) were manually counted. For PNEC quantification, whole mount SMG tissues were imaged at low magnification (10X lens, Olympus confocal). PNECs (KRT20 positive) in individual SMGs (E-cadherin positive) were counted.

#### Quantification of *SUCNR1* expression in PNECs and tuft cells

To quantify the relative *SUCNR1* expression in WT and CF pig PNECs and tuft cells, SmFISH targeting *SUCNR1*, *ASCL1*, and *POU2F3* was carried out on four tissue sections of two WT and two CF newborn pigs. Individual PNECs (*ASCL1* signals ≥ 2) and tuft cells (*POU2F3* signals ≥ 2) were imaged. Fluorescent signals (dots) were counted and recorded for *SUCNR1*, *ASCL1*, and *POU2F3* expression in individual cells. Relative *SUCNR1* expression in PNECs and tuft cells were calculated as *SUCNR1*_count_ / *ASCL1*_count_ and *SUCNR1*_count_ / *POU2F3*_count_, respectively.

#### Statistical analysis

Data points in graphs represent biological samples unless specifically indicated in the text. Results in graphs are presented as mean ± standard deviation. Statistical significance was tested using Student’s *t* test, p values less than 0.05 were considered statistically significant.

## Supplementary Material

Supplementary Material

Video S1 Video S1. Mucus in WT and CF SMGs after methacholine stimulation, Related to Figure 3. Video shows Z-stack images of two newborn WT and two newborn CF pig whole mount SMGs stained with epithelial marker (E-cadherin or EPCAM), mucin marker (WGA), and nuclei (DAPI). Gland tissues were treated with 10 μM methacholine to stimulate mucus secretion before fixation and immunostaining.

Video S2 Video S2. Succinate triggers SMG contraction, Related to Figure 4. Videos are time course stack images of three SMGs treated with 10 mM methyl succinate (left) and three SMGs treated with succinate (right). SMGs were pre-incubated with red fluorescence conjugated EPCAM antibody. Bright field and red fluorescent images were taken every 30 seconds. The time of adding methyl succinate or succinate is indicated in the videos.

Video S3 Video S3. Fura-2 based ratiometric Ca^2+^ imaging in primary myoepithelial cells, Related to Figure 7. Primary myoepithelial cells were cultured on Matrigel-coated dishes for 4 days. Cells were washed and incubated with Fura-2 (1 μm) for 30 minutes. Cells were washed and fluorescent images were taken at 340 nm and 380 nm every 3 seconds. ATP 0.1 mM was added when indicated. Ratiometric images (340/380) were generated and shown as a video.

Video S4 Video S4. ATP triggers myoepithelial contraction *in vitro*, Related to Figure 7. Primary myoepithelial cells were cultured on Matrigel coated dishes for 4 days. Cells were moved to a Ziess 880 live cell imaging system. Cells were pre-incubated with vehicle (left) or 2 μM P2Y1 receptor antagonist MRS2500 (right) for ~30 minutes. Bright field images were taken every minute for 20 imaging cycles. ATP (0.1 mM) was added to the dish at 10^th^ imaging cycle (indicated in the video).

## Figures and Tables

**Table:** KEY RESOURCES TABLE

REAGENT or RESOURCE	SOURCE	IDENTIFIER
**Antibodies**		
Mouse monoclonal anti-INSM1	Santa Cruz	sc-271408; RRID: AB_10607955
Rabbit monoclonal anti-KRT20	Boster Bio	M02828; RRID: AB_2910267
Rabbit polyclonal anti-GRP	ImmunoStar	20073; RRID: AB_572221
Rat monoclonal anti-Serotonin	Santa Cruz	sc-58031; RRID: AB_785636
Rat monoclonal anti-EPCAM	Thermo Fisher	25-5791-80; RRID: AB_1724047
Rabbit polyclonal anti-EPCAM	LSBio	LS-C700352; RRID: AB_2910268
Mouse monoclonal Anti-SYP	Santa Cruz	sc-17750; RRID: AB_628311
Rabbit polyclonal anti-SUCNR1	Novus biologicals	NBP1-00861; RRID: AB_1503315
Rabbit polyclonal anti-SUCNR1	LSBio	LS-A3315; RRID: AB_2910269
Rabbit polyclonal anti-SUCNR1	OriGene Technologies	AP01338PU-N; RRID: AB_2910270
Rabbit polyclonal anti-SUCNR1	Alomone Labs	ASR-090; RRID: AB_2756801
Mouse monoclonal anti-CGRP	Santa Cruz	sc-57053; RRID: AB_2259462
Mouse monoclonal anti-P2Y1R	Santa Cruz	sc-377324; RRID: AB_2910271
Mouse monoclonal anti-αSMA	Sigma-Aldrich	A2547; RRID: AB_476701
Rabbit polyclonal anti-KRT5	BioLegend	905501; RRID: AB_2565050
Mouse monoclonal anti-Myc tag	Thermo Fisher	MA1-21316; RRID: AB_558473
Rabbit monoclonal anti-E-cadherin	Cell Signaling	3195; RRID: AB_2291471
Mouse monoclonal anti-MUC5AC	Abcam	ab3649; RRID: AB_2146844
Rabbit polyclonal anti-MUC5B	Sigma-Aldrich	HPA008246; RRID: AB_1854203
Guinea pig polyclonal anti-VNUT	Millipore Sigma	ABN83; RRID: AB_2868445
Alexa Fluor 488 Goat anti-Rabbit IgG	Invitrogen	A-11034; RRID: AB_2576217
Alexa Fluor 568 Goat anti-Rabbit IgG	Invitrogen	A-11011; RRID: AB_143157
Alexa Fluor 647 Goat anti-Rabbit IgG	Invitrogen	A-21245; RRID: AB_2535813
Alexa Fluor 488 Goat anti-Mouse IgG	Invitrogen	A-11029; RRID: AB_2534088
Alexa Fluor 568 Goat anti- Mouse IgG	Invitrogen	A-11031; RRID: AB_144696
Alexa Fluor 647 Goat anti- Mouse IgG	Invitrogen	A-21236; RRID: AB_2535805
Alexa Fluor 647 Goat anti-Rat IgG	Invitrogen	A-21247; RRID: AB_141778
Alexa Fluor 568 Goat anti-Guinea Pig IgG	Invitrogen	A-11075; RRID: AB_2534119
**Bacterial and virus strains**		
Human SUCNR1 tagged ORF clone lentiviral particle	OriGene Technologies	RC205888L1V
**Chemicals, peptides, and recombinant proteins**		
DAPI	Invitrogen	D1306
Formaldehyde	Thermo Fisher Scientific	28908
Antifade mounting medium with DAPI	Vector Laboratories	H-1500
Wheat germ agglutinin (WGA), rhodamine	Vector Laboratories	RL-1022
WGA, Fluorescein	Vector Laboratories	FL-1021
Succinate	Sigma-Aldrich	224731
Methyl succinate	Millipore Sigma	M81101
Acetyl-β-methylcholine (MCh)	Sigma-Aldrich	A2251
ATP	Sigma-Aldrich	A6419
Porcine CGRP	GenScript	U696FFG010
5-HT (Serotonin)	Sigma-Aldrich	14927
Carbachol	Sigma-Aldrich	212385
NF-56-EJ40	MedChem Express	HY-130246
MRS2500	Tocris	2159
Ionomycin	STEMCELL Technologies	73722
Corning Matrigel, growth factor reduced	Fisher Scientific	CB-40230A
Collagenase/Hyaluronidase	STEMCELL Technologies	07912
Waymouth’s Medium	Gibco	11220035
Collagen	STEMCELL Technologies	04902
SAGM BulletKit	Lonza	CC-3118
Y-27632	STEMCELL Technologies	72302
A83-01	STEMCELL Technologies	72022
DMH-1	STEMCELL Technologies	73632
CHIR99021	STEMCELL Technologies	72052
Gentamycin sulfate	IBI Scientific	71039
Citrate Buffer (pH 6.0), Concentrate	Thermo Fisher Scientific	005000
**Critical Commercial Assays**		
ATP determination kit	Thermo Fisher	A22066
Fura-2, AM, cell permeant	Invitrogen	F1201
BioTracker 609 red Ca2+ AM dye	Sigma-Aldrich	SCT021
IHC select HRP/DAB tests	Millipore Sigma	DAB150
**Experimental models: Cell Lines**		
DMS454	Sigma-Aldrich	95062832-1VL; RRID: CVCL_2438
DMS53	ATCC	CRL-2062; RRID: CVCL_1177
**Experimental models: Organisms/strains**		
Domestic pig: wild-type & *CFTR*^−/−^	Exemplar Genetics	N/A
**Software and Algorithms**		
ImageJ	NIH	https://imagej.nih.gov/ij/
FV31S-SW	Olympus	https://www.olympus-lifescience.com/en/
Imaris	BitPlane	http://www.bitplane.com/imaris
The R Project for Statistical Computing	CRAN	https://www.r-project.org/
R Studio	R Studio	https://www.rstudio.com/
Seurat v3.1.4	Stuart et al., 2019	https://satijalab.org/seurat/

## Highlights

- PNECs localize in airway submucosal glands of humans and pigs
- Submucosal PNECs are chemosensory cells that express SUCNR1
- PNECs release ATP in response to luminal succinate
- Extracellular ATP from PNECs triggers myoepithelial cell contraction via P2Y1R
