# Supplementary material for: Pulmonary Neuroendocrine Cells Sense Succinate to Stimulate Myoepithelial Cell Contraction
Source: Dev Cell. Author manuscript; Available in PMC 2022 Dec 19. (PMC9762774; doi:10.1016/j.devcel.2022.08.010)
Supplement: Supplementary Material [file NIHMS1833894-supplement-Supplementary_Material.pdf]

Yu, Fig. S1

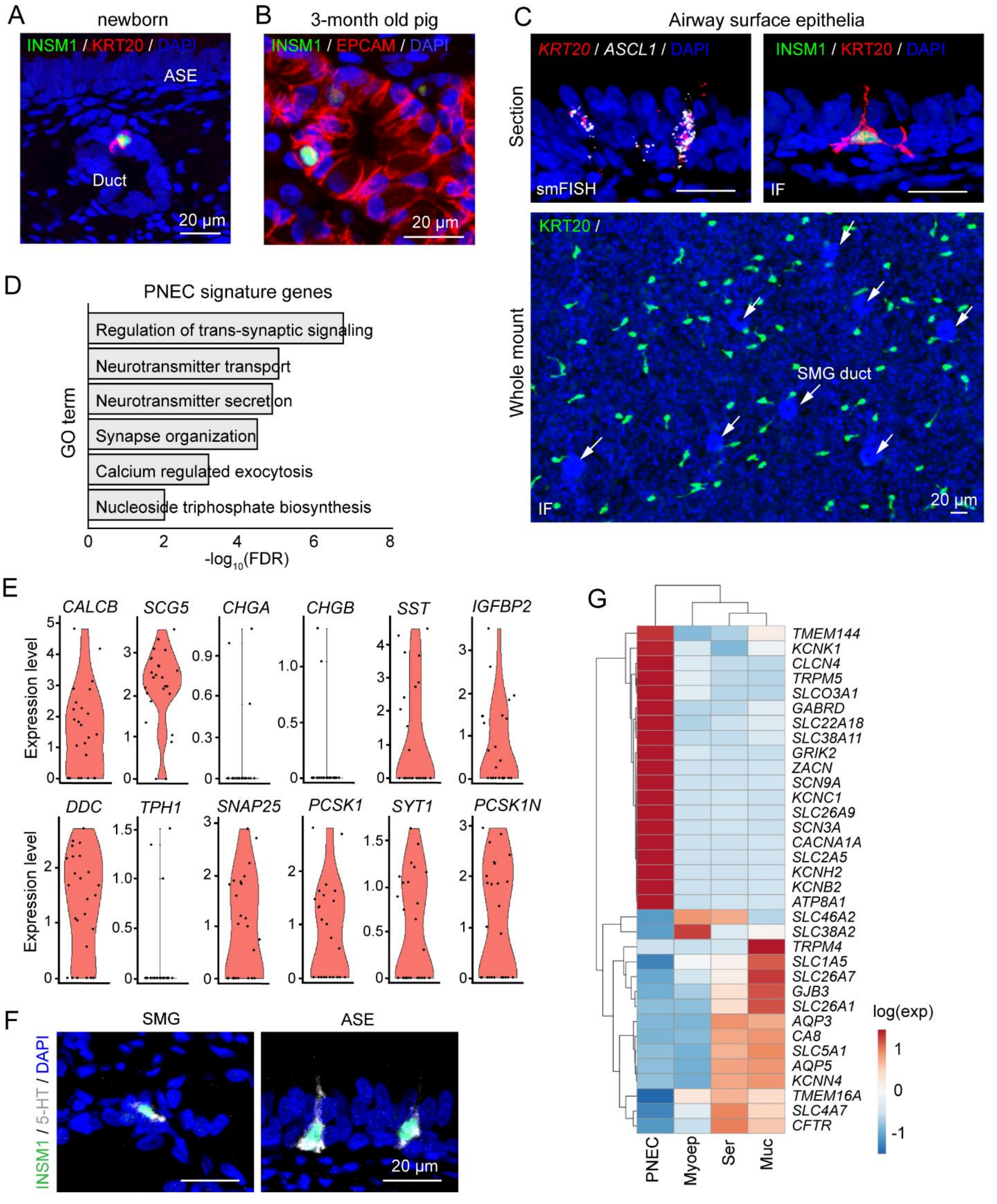
A
newborn
INSM1 / KRT20 / DAPI
ASE
Duct
20 µm
B
3-month old pig
INSM1 / EPCAM / DAPI
20 µm
C
Airway surface epithelia
Section
KRT20 / ASCL1 / DAPI
smFISH
INSM1 / KRT20 / DAPI
IF
Whole mount
KRT20 / DAPI
SMG duct
IF
20 µm
D
PNEC signature genes
GO term
Regulation of trans-synaptic signaling
Neurotransmitter transport
Neurotransmitter secretion
Synapse organization
Calcium regulated exocytosis
Nucleoside triphosphate biosynthesis
-log10(FDR)
E
Expression level
CALCB
SCG5
CHGA
CHGB
SST
IGFBP2
DDC
TPH1
SNAP25
PCSK1
SYT1
PCSK1N
F
INSM1 / 5-HT / DAPI
SMG
ASE
20 µm
G
TMEM144
KCNK1
CLCN4
TRPM5
SLCO3A1
GABRD
SLC22A18
SLC38A11
GRIK2
ZACN
SCN9A
KCNC1
SLC26A9
SCN3A
CACNA1A
SLC2A5
KCNH2
KCNB2
ATP8A1
SLC46A2
SLC38A2
TRPM4
SLC1A5
SLC26A7
GJB3
SLC26A1
AQP3
CA8
SLC5A1
AQP5
KCNN4
TMEM16A
SLC4A7
CFTR
log(exp)
PNEC
Myoep
Ser
Muc

Figure S1 | Characterization of PNECs in SMGs and airway surface epithelia, Related to Figure 1.
A) Representative image of PNECs (INSM1+ green, KRT20+ red, DAPI blue) in SMG ducts.
B) Representative images of PNECs (INSM1+, green) in acinar SMGs (EPCAM+, red) of 3-month-old pigs.
C) Representative images of smFISH and immunofluorescence (IF) staining of PNEC markers in airway surface epithelia of newborn pig sections and whole mount tissues.
D) Gene ontology (GO) analysis of PNEC signature genes. FDR, false discovery rate.
E) Expression of genes that are involved in secretory function in porcine SMG PNECs. Upper panel, genes for neurotransmitters and neuropeptides; lower panel, genes involving in neurotransmitter processing.
F) Serotonin (5-HT, gray) in SMG and airway surface PNECs (INSM1+). ASE, airway surface epithelia.
G) Heatmap of PNEC signature transporters and representative transepithelial electrolyte transporters in SMGs. Ser, serous cell; Myoep, myoepithelial cell; Muc, mucous cell.

Yu, Fig. S2

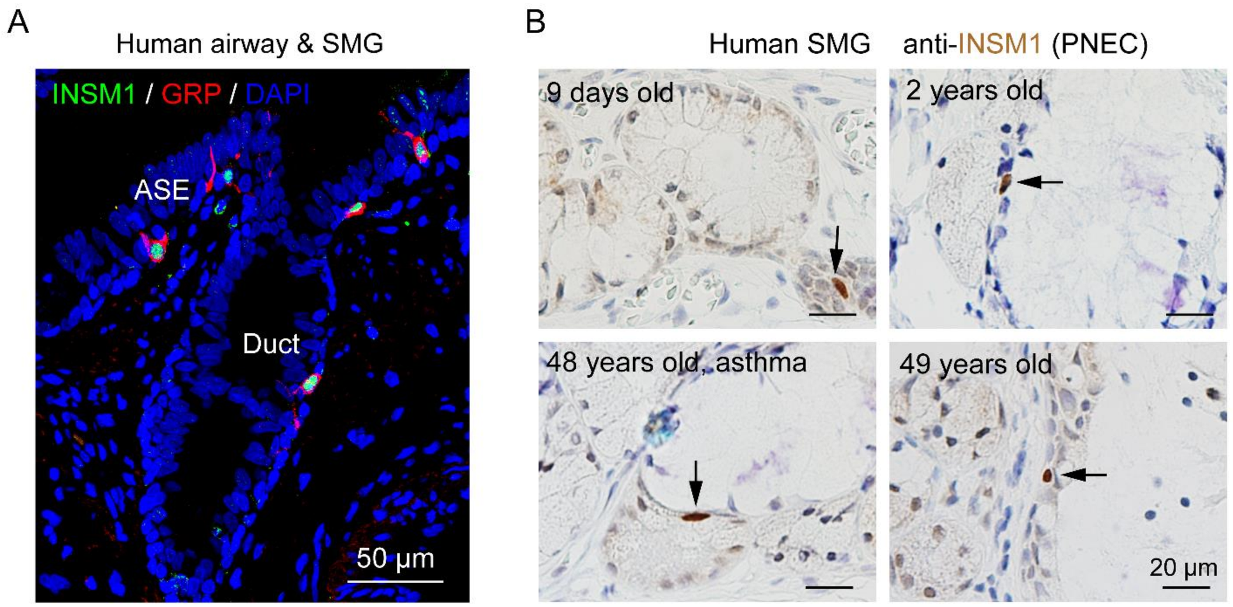

Figure S2 | Characterization of PNECs in human SMGs, Related to Figure 1.
A). Immunofluorescence of PNEC markers (INSM1 & GRP) in human airways and SMGs. ASE, airway surface epithelia.
B). PNECs (INSM1+, arrows) in human airway SMGs. The age and disease of the human donor lungs are shown.

Yu, Fig. S3

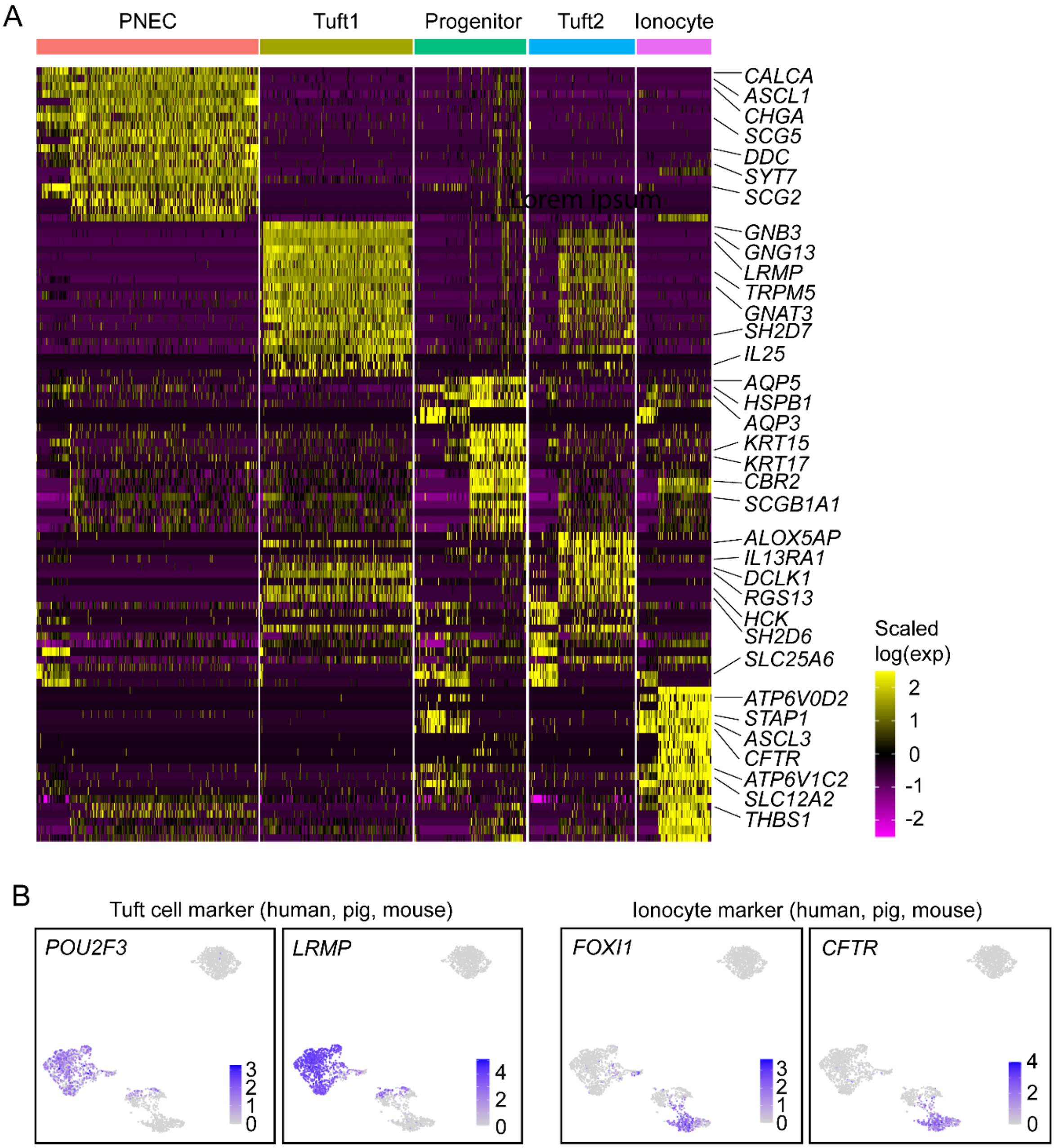

Figure S3 | Gene expression pattern in rare cell types, Related to Figure 2.
A) Heatmap of top 20 markers for rare cell types in human, pig, and mouse airway scRNA-seq datasets. Cell type names were assigned based on known markers for that cell type.
B) Expression of tuft cell and ionocyte markers in uniform manifold approximation and projection (UMAP).

Yu, Fig. S4

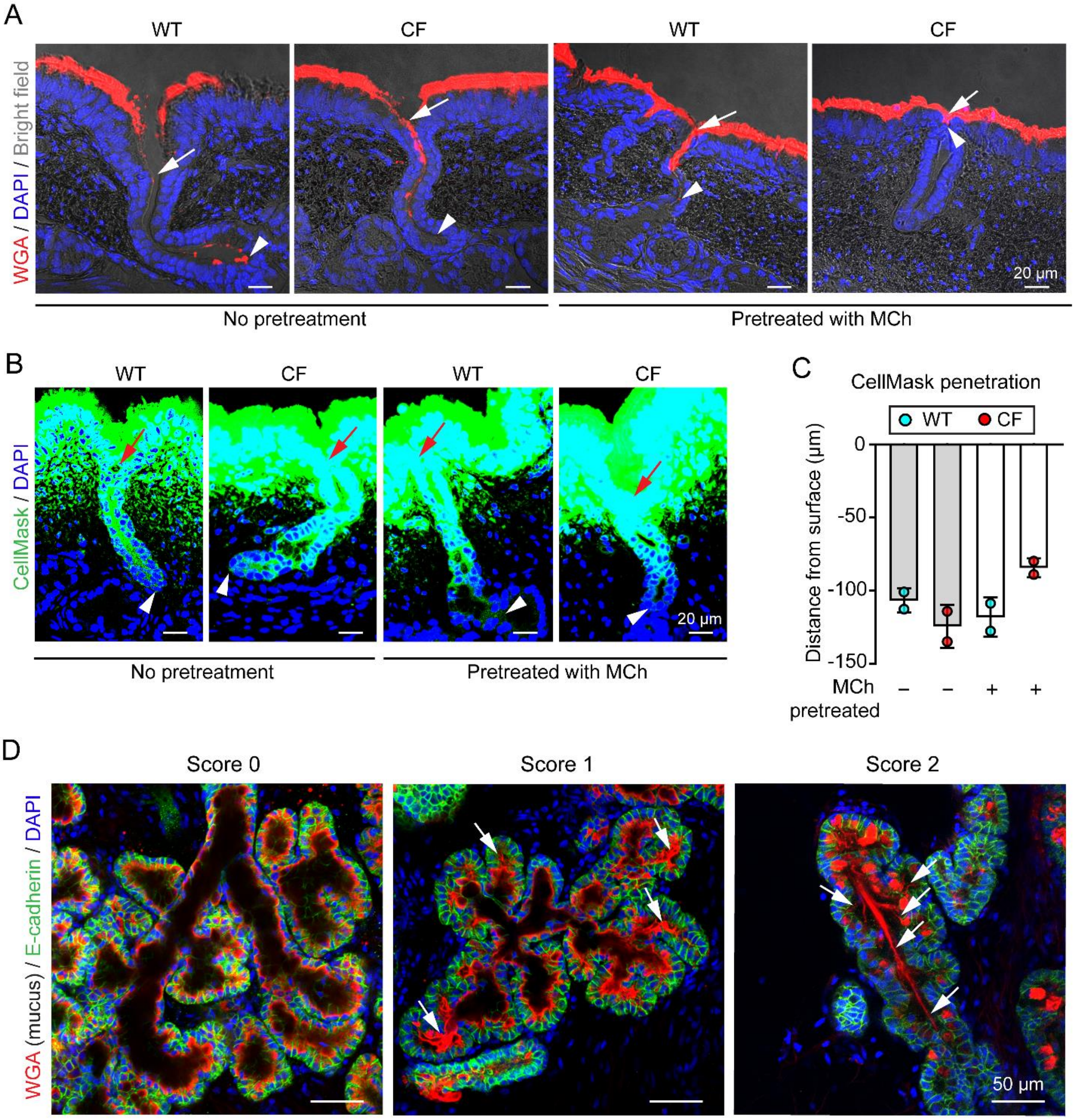
A
WT
CF
WT
CF
WGA / DAPI / Bright field
20 µm
No pretreatment
Pretreated with MCh
B
WT
CF
WT
CF
CellMask / DAPI
20 µm
No pretreatment
Pretreated with MCh
C
CellMask penetration
WT
CF
Distance from surface (µm)
0
-50
-100
-150
MCh pretreated
–
–
+
+
D
Score 0
Score 1
Score 2
WGA (mucus) / E-cadherin / DAPI
50 µm

Figure S4 | Mucus obstruction reduces molecule diffusion in CF SMGs, Related to Figure 3.
A, B) Examples of rhodamine-WGA (**A**) and CellMask (**B**) diffusion into SMGs of WT and CF pigs. Tracheal segments were treated with methacholine (10 μM) for 10 minutes, washed with Hank's balanced salt solution 3*10 minutes before starting the diffusion assay. Arrows point to the SMG ducts at the airway surface; arrowheads point to the WGA or CellMask signals deep in SMG ducts.
C) Summary of distance that CellMask (50 μM) penetrating down SMG lumen.
D) Representative images of MUC5B mucin staining (WGA, red) in SMGs (E-cadherin+). Mucus scores were assigned to individual SMGs based on the abundance of mucus in the lumen of SMGs. Score 0, no mucus; score 1, small amount of attached mucus (arrows); score 2, mucus strands across most of the lumen. The images from 'Score 2' in Figure S4D and 'CF' in Figure 3D are different image layers from the same SMG.

Yu, Fig. S5

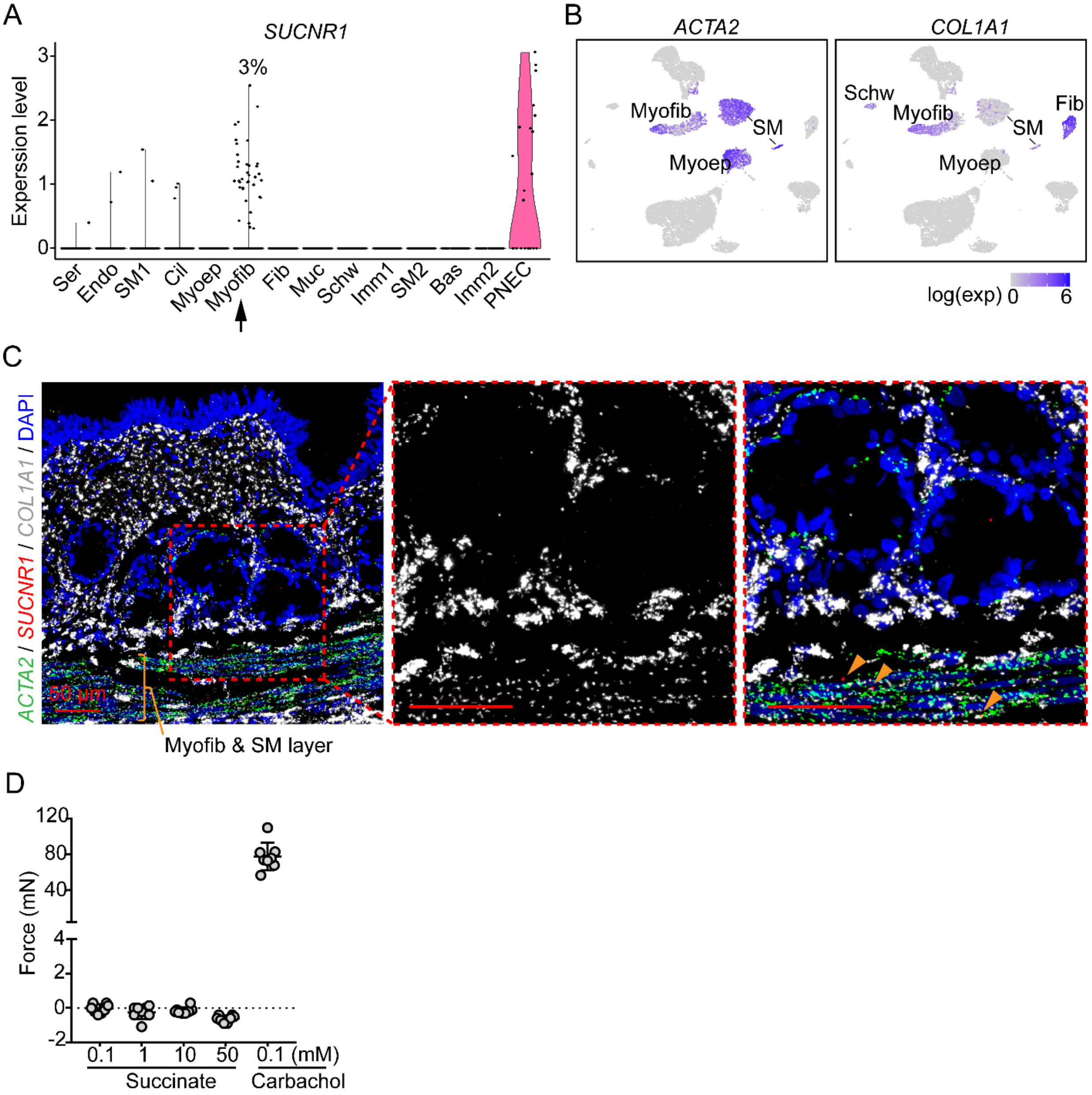
A
SUCNR1
3%
Expression level
3
2
1
0
Ser
Endo
SM1
Cil
Myoep
Myofib
Fib
Muc
Schw
Imm1
SM2
Bas
Imm2
PNEC
B
ACTA2
COL1A1
Myofib
SM
Myoep
Schw
Fib
log(exp) 0
6
C
ACTA2 / SUCNR1 / COL1A1 / DAPI
50 µm
Myofib & SM layer
D
Force (mN)
120
80
40
4
2
0
-2
0.1
1
10
50
0.1 (mM)
Succinate
Carbachol

Figure S5 | Succinate does not induce myofibroblast contraction, Related to Figure 4.
A) Plot of *SUCNR1* expression in SMG scRNA-seq data grouped by cell type (Yu et al., 2022). Arrow points a small proportion (~3%) of myofibroblasts expressing *SUCNR1*. Ser, serous cell; Endo, endothelial cell; SM, smooth muscle cell; Cil, ciliated cell; Myoep, myoepithelial cell; Myofib, myofibroblast; Fib, fibroblasts; Muc, mucous cell; Schw, Schwann cell; Bas; basal cell; Imm, immune cell.
B) Expression pattern of *ACTA2*, and *COL1A1* (Type 1 collagen, α1) in SMG scRNA-seq data grouped by cell type.
C) Representative images of smFISH of *ACTA2, SUCNR1* and *COL1A1* in SMGs and surrounding tissues. Note that no *ACTA2*+*COL1A1*+ cells (myofibroblasts) surround SMGs. A few cells (arrowheads) from the smooth muscle (SM) and myofibroblast (myofib) layer are stained for *SUCNR1*.
D) Smooth muscle and myofibroblast related tracheal contraction. 8 tracheal rings from 2 pigs were sequentially treated with different concentrations of succinate and carbachol. Values at the peak of each condition were recorded and included for analysis.

Yu, Fig. S6

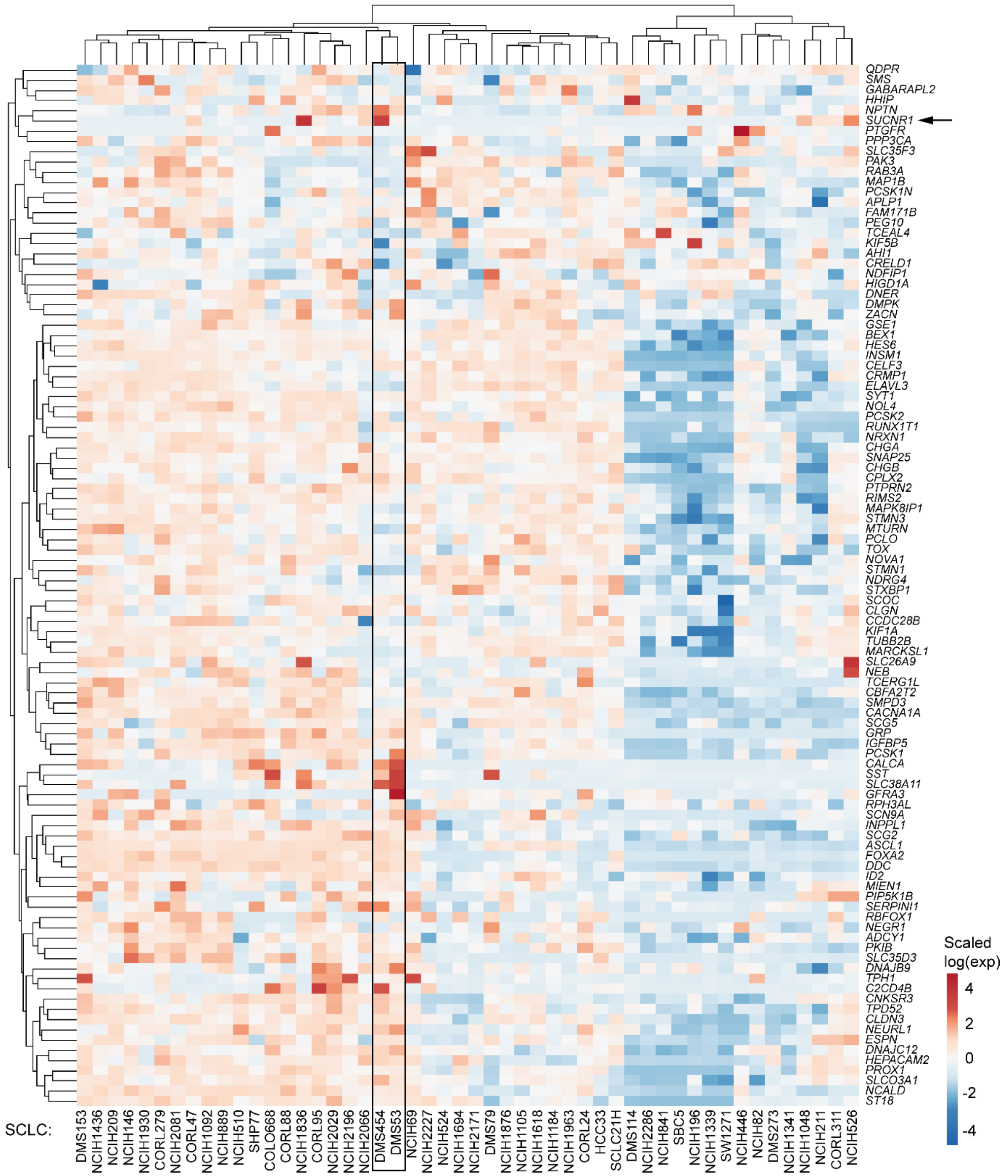

Figure S6 | Characterization of PNEC-like small cell lung cancer cell lines, Related to Figure 5.
Heatmap of PNEC marker gene expression in published RNA sequencing of small cell lung cancer (SCLC) cell lines (Ghandi et al., 2019). The columns for DMS454 and DMS53 cells are highlighted; arrow on right points to *SUCNR1*.

Yu, Fig. S7

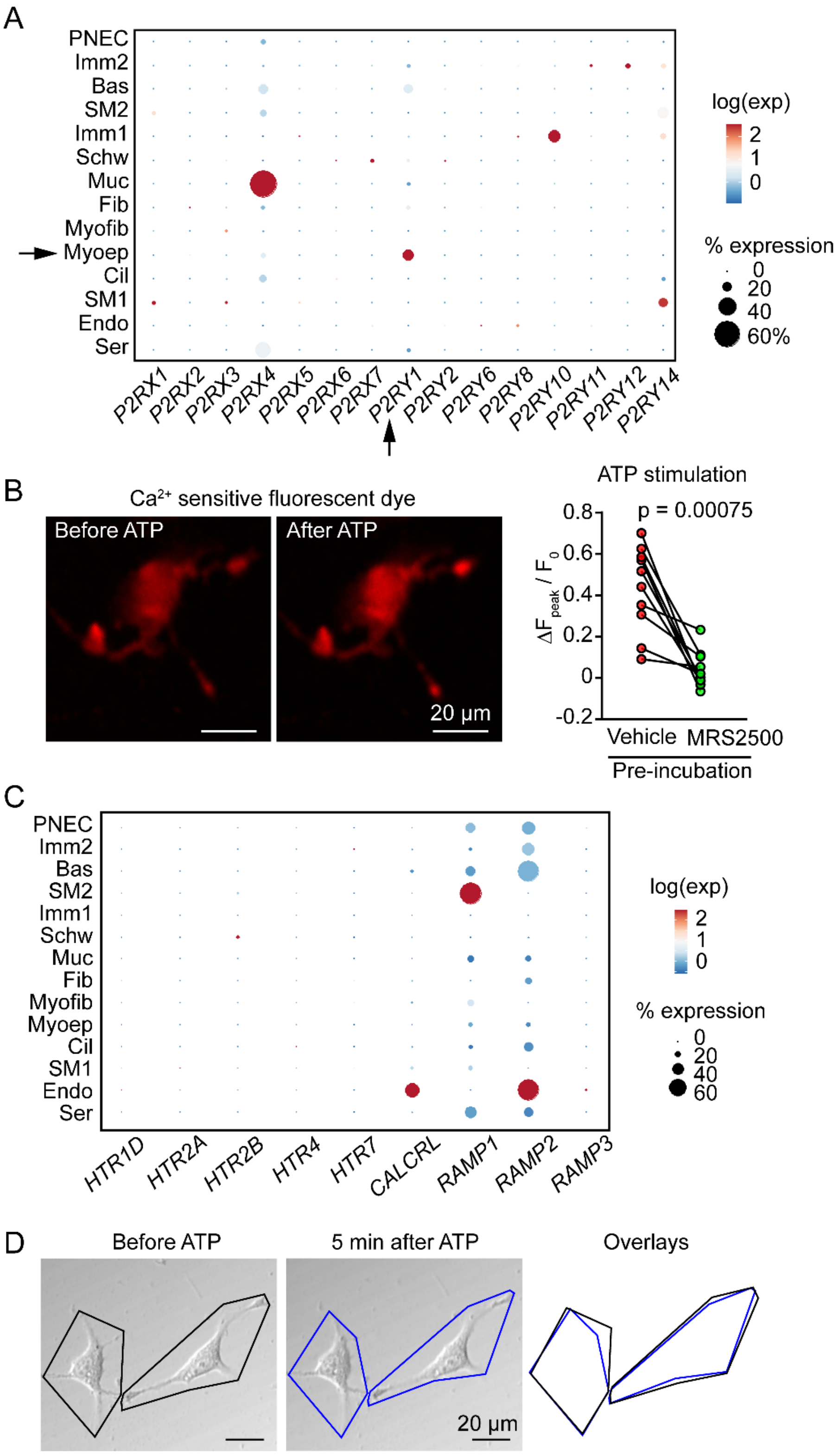
A
PNEC
Imm2
Bas
SM2
Imm1
Schw
Muc
Fib
Myofib
Myoep
Cil
SM1
Endo
Ser
P2RX1
P2RX2
P2RX3
P2RX4
P2RX5
P2RX6
P2RX7
P2RY1
P2RY2
P2RY6
P2RY8
P2RY10
P2RY11
P2RY12
P2RY14
log(exp)
2
1
0
% expression
0
20
40
60%
B
Ca²⁺ sensitive fluorescent dye
Before ATP
After ATP
20 µm
ATP stimulation
p = 0.00075
ΔF peak / F 0
0.8
0.6
0.4
0.2
0
-0.2
Vehicle
MRS2500
Pre-incubation
C
PNEC
Imm2
Bas
SM2
Imm1
Schw
Muc
Fib
Myofib
Myoep
Cil
SM1
Endo
Ser
HTR1D
HTR2A
HTR2B
HTR4
HTR7
CALCRL
RAMP1
RAMP2
RAMP3
log(exp)
2
1
0
% expression
0
20
40
60
D
Before ATP
5 min after ATP
Overlays
20 µm

Figure S7 | Myoepithelial cells respond to ATP via P2Y1 receptors, Related to Figure 7.
A) Expression pattern of purinergic genes in SMG scRNA-seq data (Yu et al., 2022) grouped by cell type. P2X4 receptor (*P2RX4*), an apical expressed purinoceptor for ATP, is not accessible for basolateral ATP from PNECs. Myoepithelial distribution of P2Y1 receptor (*P2RY1*) makes it an interesting candidate for basolateral ATP from SMG PNECs.
B) Representative images and summary of Ca^{2+} imaging in primary myoepithelial cells responding to ATP. Cells were labeled with a Ca^{2+} sensitive dye (BioTracker 609 Red Ca^{2+} AM Dye, Millipore sigma #SCT021). They were treated with or without P2Y1 receptor antagonist MRS2500 (2 μM) before Ca^{2+} imaging. Each data point represents the mean of imaged cells from a pig; $p = 0.00075$, paired Student's *t* test.
C) Expression pattern of 5-HT and CGRP receptors in SMG scRNA-seq data (Yu et al., 2022) grouped by cell type.
D) Bright field images of primary myoepithelial cells before and 5 minutes after ATP addition. Closed lines connect branch tips of individual myoepithelial cells (defined as total area). Overlays highlight reduction of total area after ATP treatment.

Table S1. PNEC-specific G protein-coupled receptors, Related to Figure 2A.

Gene	p val	avg logFC	pct.1	pct.2	FDR	Mean expression
SUCNR1	0	1.529	0.48	0.003	0	1.537
GABBR2	0	0.399	0.2	0	0	0.399
PTGFR	3.89E-115	1.670	0.72	0.023	7.62E-111	1.711
GPR27	1.32E-10	0.329	0.28	0.037	2.59E-06	0.387
GPR143	1.38E-08	0.652	0.56	0.192	0.000270	0.824
CELSR2	0.001359	0.261	0.2	0.058	1	0.321
CELSR1	0.002409	0.408	0.32	0.134	1	0.534
GPRC5C	0.006642	0.467	0.32	0.151	1	0.662
ADGRL1	0.031497	0.324	0.32	0.184	1	0.543
TPRA1	0.100434	0.375	0.28	0.173	1	0.593

Abbreviation: p val, p value; avg logFC, mean fold change at natural log scale; pct.1, percentage of positive gene expression in PNECs; pct.2, percentage of positive gene expression in non-PNECs; FDR, false discovery rate.

Table S2. Myoepithelial cell specific receptors, Related to Figure 6A.

Gene	p val	avg logFC	pct.1	pct.2	FDR	Mean expression
NTRK3	0	1.357	0.664	0.028	0	1.44
FGFR1	0	1.044	0.598	0.116	0	1.27
CXCR4	0	0.979	0.616	0.136	0	1.49
LGR6	0	0.469	0.213	0.012	0	0.48
EDAR	1.33E-210	0.266	0.121	0.006	2.60E-206	0.27
P2RY1	9.45E-195	0.534	0.247	0.041	1.85E-190	0.57
FZD1	2.60E-149	0.637	0.387	0.133	5.10E-145	0.8
IL1RAP	2.93E-117	0.546	0.333	0.114	5.74E-113	0.72
FZD7	2.98E-80	0.419	0.244	0.084	5.83E-76	0.53
F2RL1	2.99E-59	0.457	0.328	0.168	5.86E-55	0.68
TRAF5	3.04E-57	0.286	0.156	0.047	5.95E-53	0.37
RARG	1.43E-41	0.366	0.228	0.11	2.80E-37	0.49

Abbreviation: p val, p value; avg logFC, mean fold change at natural log scale; pct.1, percentage of positive gene expression in PNECs; pct.2, percentage of positive gene expression in non-PNECs; FDR, false discovery rate.

Table S3. Antibodies, single-molecule fluorescence in situ hybridization probes, and qRT-PCR primers, Related to STAR Methods.

Primary antibody information

**Name**	**cat #**	**Ventor**	**Dilution**	**Tested species**	**Section type**	**IHC or IF**
Anti-INSM1	sc-271408	SANTA CRUZ	1:200	Human, pig	Cryosection & FFPE	IF & IHC
Anti-KRT20	M02828	BOSTER	1:500	Pig	Cryosection & FFPE	IF & IHC
Anti-5-HT	sc-58031	SANTA CRUZ	1:100	Pig	Cryosection	IF
Anti-EPCAM	50-108-70	Thermo Fisher	1:100	Pig	Live tissue	IF
Anti-EPCAM	LS C700352	LSBio	1:100	Pig	Cryosection & whole tissue	IF
Anti-SYP	sc-17750	SANTA CRUZ	1:200	Pig	Cryosection	IF
Anti-SUCNR1	NBP1-00861	Novus biologicals	1:200	Human, pig	Cryosection & FFPE	IF
Anti-SUCNR1	LS-A3315-50	LSBio	1:200	Human, pig	Cryosection & FFPE	IF
Anti-SUCNR1	AP01338PU-N	OriGene Technologies	1:200	Human, pig	Cryosection & FFPE	IF
Anti-SUCNR1	ASR-090	Alomone Labs	1:200	Human, pig	Cryosection & FFPE	IF
Anti-CGRP	sc-57053	SANTA CRUZ	1:100	Human	FFPE	IF
Anti-P2Y1R	sc-377324	SANTA CRUZ	1:100	Pig	FFPE	IF
Anti-αSMA	A2547	Sigma-Aldrich	1:100	Pig	Cryosection	IF
Anti-KRT5	905501	BioLegend	1:500	Pig	Cryosection	IF
Anti-Myc tag	MA1-21316	Thermo Fisher	1:500	Human	Cryosection	IF
Anti-E-cadherin	3195	Cell Signaling	1:200	Pig	Whole tissue	IF
Anti-MUC5AC	ab3649	Abcam	1:1000	Human	FFPE	IF
Anti-MUC5B	HPA008246	Sigma-Aldrich	1:200	Human	FFPE	IF

Secondary antibody information

Thermo Fisher	Alexa Fluor 488	Alexa Fluor 568	Alexa Fluor 647
Goat anti-rabbit IgG antibody conjugate	1:500 ~ 1:1000	1:500 ~ 1:1000	1:500 ~ 1:1000
Goat anti-mouse IgG antibody conjugate	1:500 ~ 1:1000	1:500 ~ 1:1000	1:500 ~ 1:1000
Goat anti-rat IgG antibody conjugate			1:500 ~ 1:1000

Single-molecule fluorescence in situ hybridization probes

Pig Gene	mRNA sequences targeted by paired probes
ASCL1	GTCGGACGAGGGCTCTTACGACCCGCTCAGCCCA
	AACCAACCTCTATGCCAACTAAGCGTCACATGCGAAGC
	CTCCAACCATTCACGGAGATAGAAAGAGTGACTAGGGCCT
	TGTGCTATACTCAGTCCTTTAAAGCCCCAATTGGTGG
KRT20	TTCTAGACGCGTGGTGAGCTATGGGAGCGACCAAGCTGAA
	GAGGAGGAAGTGCAGAGTCTACGCGCTCATCTGGGCAATA
	GGAGGAAACCAAAGCTCGTTATGGTGCCTATCTGGCCAAA
	TGGTGCAGATTCGGACCGATACAGAACACCAGATCAATGA
SUCNR1	TATGGACACATGCTCTGCATAAGCAACCGATACGTGCTTC
	TTTTATCAGCATTGATCGATACATTCTCATGAAGTATCCT
	ATTTGGGGTTTAGTAACTGTAGAGCTCCTGCCCATACTTC
	CTGTGAATAGCACCAGCTGTACTGATTATGCGAGTTCTGG
POU2F3	GCACCATGCCTGGAACAGTAACGTCATCCTGTTCCCCTG
	CTGTGATTGAACCGTGTGCTAACTCCTAGTGCTCTTGAAA
	CTGCGGCCGTGGTCTTTTTAGGAAGGACCTCTTGCCATT
	ACAATCTCCAAACAGATTCTAGACTCTGGAAAGATTTGCA
SLC17A9	CTCTGGGTGTGGTACGTGTACAAGTGCCTGCTGAGCGA
	CCACCTCATCAATCAGGGTTATAGGACCATCTCCGTGCGG
ACTA2	CGTCCATTGTGGGACGTCCTAGACATCAGGGGGTGATGGT
	CACCACTCTTTCTACAATGAGCTTCGTGTTGCCCCAGAAG
	GATCATTGCCCCTCCTGAGCGCAAATACTCCGTCTGGA
PCP4	ATCCCGGAGCTGCCGTTACAACATCATGAGTGAGCGA
	CCATCATCTGTCAAAAAATTAAAAAAAGCAACACCCTTGA
	GCATGTACAGAGACCGTGCTATTTATACCCTGGTAGGAAG
P2RY1	GGCCCCAGAGCCGAAGTACTGGCATGCAAGGTAGG
	CCAGTCCAGTTCGGTCATAGATCGGGACGGATTCATGG
	TCCCGTTCAAATGCGCGCTTACCAAGACAGGCTTCCAATT
	GCATCAGTGCGCACCGGTACAGCGGTGTGGTGTACCCT
COL1A1	CACGCATGAGCGGACGCTAACCCCCACCCCAGCCG
	CGTACAGAACGGCCTCAGGTACCATGACCGAGACGTGTGG
	CTTTGCTCCCCAGTTGTCTTATGGCTATGATGAGAAGTCA

qRT-PCR primers		
**Human Gene**	**Forward**	**Reverse**
RPL13A	GGCCCCTACCACTTCCG	ACTGCCTGGTACTTCCA
SUCNR1	GGAGACGTGCTCTGCATAAG	AGGTGTTCTCGGAAAGGATACTT
SLC17A9	CACCTCGGGGATCGGATTG	GCAGGGAAGTAAACCCCTTGG
